# Brain clearance of protein aggregates: a close-up on astrocytes

**DOI:** 10.1186/s13024-024-00703-1

**Published:** 2024-01-16

**Authors:** Veronica Giusti, Gurkirat Kaur, Elena Giusto, Laura Civiero

**Affiliations:** 1 IRCCS San Camillo Hospital, Venice, Italy; 2 Department of Biology, University of Padova, Padua, Italy

**Keywords:** Amyloid proteins, Astrocytes, Clearance, α-Synuclein, Tau

## Abstract

Protein misfolding and accumulation defines a prevailing feature of many neurodegenerative disorders, finally resulting in the formation of toxic intra- and extracellular aggregates. Intracellular aggregates can enter the extracellular space and be subsequently transferred among different cell types, thus spreading between connected brain districts.

Although microglia perform a predominant role in the removal of extracellular aggregated proteins, mounting evidence suggests that astrocytes actively contribute to the clearing process. However, the molecular mechanisms used by astrocytes to remove misfolded proteins are still largely unknown.

Here we first provide a brief overview of the progressive transition from soluble monomers to insoluble fibrils that characterizes amyloid proteins, referring to α-Synuclein and Tau as archetypical examples. We then highlight the mechanisms at the basis of astrocyte-mediated clearance with a focus on their potential ability to recognize, collect, internalize and digest extracellular protein aggregates. Finally, we explore the potential of targeting astrocyte-mediated clearance as a future therapeutic approach for the treatment of neurodegenerative disorders characterized by protein misfolding and accumulation.

**Supplementary Information:**

The online version contains supplementary material available at 10.1186/s13024-024-00703-1.

## Background

**Fig. 1:**
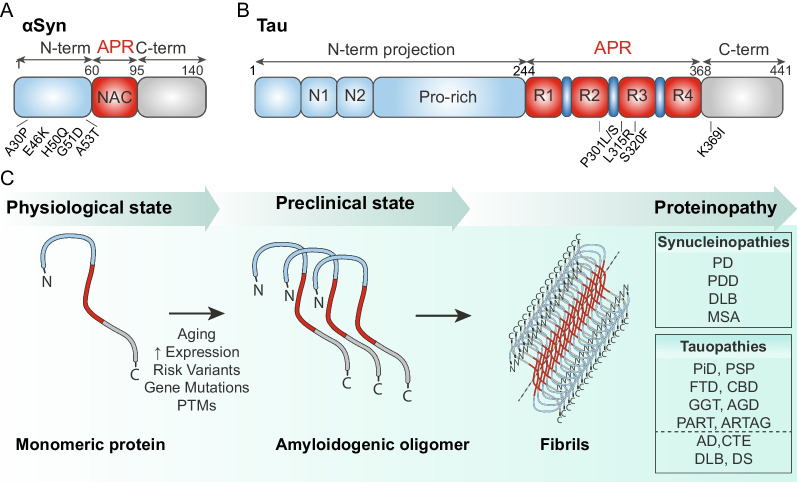
Common processes of α-Syn and Tau aggregation in neurons. Schematic representation of α-Syn (**A**) and Tau (**B**) structures with depicted some of the most common mutations. Both the proteins include an aggregation prone region (APR), a short sequence of amino acids (6–7 residues) which confers an amyloid-like competence to form β-sheet structures. (**C**) In physiological conditions amyloidogenic proteins exist mainly as monomers. However, several factors may contribute to the progressive formation of oligomers and filaments, finally leading to the clinical onset of proteinopathies. Depending on the patient characteristic, α-Syn and Tau can give origin to a spectrum of synucleinopathies and tauopathies, respectively

Proteinopathies of the central nervous system (CNS) are characterized by the progressive misfolding, aggregation and accumulation of amyloidogenic proteins, which ultimately results in the formation of pathological deposits [1]. The amyloidogenic nature of these proteins is defined by their propensity to polymerize into fibrils with a predominant cross β-sheet structure, most likely due to the presence of distinctive aggregation prone regions (APRs), in spite of otherwise dissimilar sequences, structures and functions [2] (Fig. 1A-B). Among the most studied, Amyloid-β (Aβ), Tau, α-Synuclein (α-Syn) and TAR DNA-binding protein-43 are commonly associated, either alone or in combination, with some major neurodegenerative disorders (NDs), including Alzheimer disease (AD), Parkinson’s disease (PD) and amyotrophic lateral sclerosis (for a comprehensive review, please see [3]).

α-Syn and Tau are prototypical examples of amyloidogenic proteins, for which similarities in the course of misfolding, aggregation and spreading have been described, thus supporting their implication in the onset of major proteinopathies [4, 5]. α-Syn consists of three distinct regions: an amphipathic acetylated N-terminal domain (aa 1–60), the non-amyloid β component (NAC) domain (aa 61–95) and an acidic carboxyl tail (aa 96–140) (Fig. 1A). The NAC domain represents the core of the protein, and it is accounted as the major responsible for α-Syn-induced toxicity, due to its potential to promote aggregation [6]. α-Syn aggregates are primarily found within Lewy bodies and Lewy neurites in neurons of patients affected by PD, PD with dementia and dementia with Lewy bodies (DLB), a class of proteinopathies with similar clinical outcomes, characterized by substantial cognitive and motor alterations, collectively indicated as “synucleinopathies” [7]. Of note, α-Syn aggregates are not a peculiar characteristic of neurons, although most studies so far have analyzed the toxicity of α-Syn inclusions mainly in these cells. Indeed, the presence of α-Syn aggregates has been confirmed also in glial cells. An example is represented by multiple system atrophy (MSA), in which α-Syn is predominantly accumulated within oligodendrocytes [8].

Similarly, the expression “tauopathies” has been introduced to indicate a group of proteinopathies defined by the presence of Tau aggregates. Tau is encoded by the microtubule-associated protein Tau (MAPT) gene, which can give rise to different isoforms of the protein upon multiple alternative splicing events. Specifically, Tau isoforms can be distinguished for the presence of either three (3R) or four (4R) repetitions of highly conserved domains at their C-terminal combined with the expression of none (0 N), one (1 N) or two (2 N) repeated sequences at the N-terminal [9] (Fig. 1B). In some cases, specific isoforms can be associated to distinct tauopathies; as an example, 3R-Tau aggregates are commonly found in patients affected by Pick’s disease, 4R-Tau is characteristic of progressive supranuclear palsy (PSP) tangles, while AD patients present aggregates with both 3R- and 4R-Tau isoforms [10]. Moreover, tauopathies are conventionally indicated as “primary” when dysfunctional Tau is the leading cause of the disease, and “secondary” when Tau-pathology is contingent on (or associated with) other major pathological conditions [11]. Primary tauopathies include frontotemporal dementia, Pick’s disease, PSP, corticobasal degeneration (CBD), globular glial tauopathy, argyrophilic grain disease, primary age-related tauopathy and aging-related Tau astrogliopathy, while AD, chronic traumatic encephalopathy, Down syndrome and dementia with Lewy bodies belong to secondary tauopathies [12].

Amyloid aggregates (with a major exception for Aβ) are mainly retained within the cytoplasm of affected cells; in some cases, however, they can be transferred to surrounding cells or released in the extracellular space, thus triggering the progression and spreading of the pathology. It has been consistently shown that extracellular aggregates can alter neurotransmitter signaling, impair synaptic transmission and long-term potentiation (LTP) [13]. As an example, the application of α-Syn oligomers reduces LTP in mice hippocampal slices through the activation of N-methyl-D-aspartate receptors [13]. Likewise, the increased phagocytic activity performed by glial cells in presence of extracellular aggregates can lead to detrimental effects, finally affecting their homeostatic functions and adding to the overall degree of inflammation [14]. Indeed, the treatment of rat dopaminergic primary neurons with α-Syn in the presence of microglia induces neuronal toxicity, most likely due to an increased oxidative stress mediated by the activated microglia [15]. Therefore, the proper removal of extracellular aggregates is crucial to preserve a viable microenvironment and prevent CNS damage.

Microglia are commonly indicated as the tissue-resident macrophages, representing the forefront in the removal of unwanted debris, including protein aggregates. However, it is becoming increasingly obvious that astrocytes can offer a significant help in this clearance activity and may compensate for impaired microglial phagocytosis, as suggested by in vivo experimental models of microglia-directed ablation [16]. Moreover, astrocytes actively participate in the elimination of unwanted, toxic material, including aggregates, and their engagement in synaptic pruning in the developing brain is well established [17]. In support of this, astrocytes express several receptors, cytosolic and transmembrane molecules involved in phagocytosis and macropinocytosis, some of which are shared with microglia. Among these AXL, MER Proto-Oncogene Tyrosine Kinase (MERTK), Multiple EGF Like Domains 10 (MEGF10), αvβ5 integrin, the low density lipoprotein receptor-related protein 1 (LRP1), Cone-Rod Homeobox, Dedicator Of Cytokinesis 1 and GULP PTB domain containing engulfment adaptor 1 (Gulp1) have been shown to be expressed in mice [17, 18]. Interestingly, the expression of some of these receptors may be upregulated in mice upon prolonged stress or in pathological conditions such as experimental transient middle cerebral artery occlusion, a model of stroke [19, 20].

Nevertheless, questions related to the nature and specificity of the molecular machinery responsible for amyloid proteins recognition and internalization in astrocytes remain largely unsolved. How do astrocytes collect extracellular aggregates? Is this a process mediated by receptors able to differentially recognize distinct amyloid proteins, or is it a less targeted process involving common players? Are mechanisms other than phagocytosis implicated? In addition, there are several questions associated with the outcome of aggregates upon internalization: are astrocytes able to efficiently digest the engulfed material, or is their degradative system unprepared to fully process amyloid aggregates? In the latter case, how are the aggregates finally cleared out? Answers to these questions will allow us to better define the still questionable role of astrocytes in NDs (beneficial vs detrimental) and to possibly find novel and specific therapeutic targets to restore or enhance astrocyte-mediated clearance.

## Mechanisms for α-Syn and Tau aggregation and propagation

In homeostatic conditions, both α-Syn and Tau exist mainly in the form of soluble proteins. However, in pathological circumstances, a yet unidentified triggering event initiates a cascade of protein misfolding and oligomerization, which leads to the formation of “seeds”. For the most part, these seeds are promptly recognized and halted by the cellular quality control system which allows the elimination of misfolded or dysfunctional proteins and ensures cellular proteostasis [21]. Clearance of altered proteins may occur either through the boost of the intracellular degradation systems or through the release of the damaged proteins in the extracellular space. Protein degradation can follow two different pathways: the ubiquitin-proteasome system (UPS), mainly addressed to the elimination of short-lived and soluble proteins, or the lysosomal system, which is mainly responsible for the degradation of long-lived and insoluble aggregates [22]. The UPS is a multistep process, which involves the activity of three different ubiquitin enzymes and of the proteasome, a protein complex dedicated to the catalytic degradation of ubiquitin-marked proteins [23]. Lysosomes, instead, receive proteins determined for elimination from different vesicular pathways, including endosomes, phagosomes, autophagosomes and amphisomes. While endosomes and phagosomes are responsible for the internalization of extracellular material, amphisomes and autophagosomes are engaged in the clearance of intracellular misfolded or dysfunctional proteins, a process known as autophagy -further divided into microautophagy, macroautophagy and chaperone-mediated autophagy (for a detailed review, please see [24]). Macroautophagy starts with the formation of a newly synthesized double-layered membrane, called phagophore, which encloses the material to be degraded and subsequently fuses to form an autophagosome. The latter can further coalesce with late endosomes or multi-vesicular bodies to form amphisomes, which represent a junction point between endocytosis and autophagy and contribute to the maturation of autophagosomes [25]. Finally, amphisomes fuse with lysosomes to form autolysosomes, where the cargo is finally degraded by lysosomal enzymes [25].

Otherwise, to maintain their own homeostasis, cells may release dysfunctional proteins into the extracellular space either as free molecules or by means of exosomes; moreover, cells can transfer some of their content to neighboring cells through the extension of tunneling nanotubes (TNTs) (see below).

Despite the availability of these options for the cells to remove misfolded proteins, in a minority of cases, seeds may persist and initiate a nucleation event, offering the basis for the progressive maturation of amyloid fibrils. Fibrils can in turn break and serve as new seeds, thus perpetuating a deleterious process, which finally results in neuronal dysfunction and death (Fig. 1C) [1]. Along this multi-step progression, aberrant proteins assume several transient conformations, and are gradually converted into β-sheet-rich oligomers, thus far considered the most likely species at fault for the onset and development of the pathological insult [26]. The chance for the oligomer formation to occur increases significantly with aging or in pathological circumstances characterized by elevated expression, mutations, or post-translational modifications (PTMs) of native proteins [27–30]. Indeed, studies of genetic imbalance and comparative genome wide association studies focusing on alterations within the promoter region have shown that variabilities in the amount of protein expression correlate with the formation of aggregates and the consequent onset of NDs [31–33]. Likewise, gene mutations, as well as protein PTMs, may also result in the formation of fibrils with specific structural and functional properties, also known as strains (see Suppl. Table 1). Initial studies have shown that mutations such as A53T and A30P lead to faster kinetics of fibrillogenesis in vitro and in cells compared to wild type α-Syn [34, 35]. Moreover, mice overexpressing A53T show neuropathological signs typical of PD, whose severity correlates with the amount of α-Syn expressed [36]. Boyer and colleagues have shown that the H50Q mutation of α-Syn determines a rearrangement of the protofilament interface, finally leading to a fibril with faster kinetics of fibrillogenesis in vitro, which resulted in higher seeding and toxicity in cells [37]. Zhao et al. have shown that the E46K mutation determines a conformational change by altering some electrostatic interactions with residue K80 of α-Syn, thus leading to a structural polymorph with less stability [38]. Sun and colleagues have demonstrated that α-Syn fibrils with the G51D mutation can induce wild type fibrils to change their structural organization and cross seed with mutant fibrils in vitro [39]. N- and C-truncated polymorphs are also known to exist as a result of α-Syn incomplete degradation. Notably, C-truncated α-Syn exhibits a higher propensity to aggregate and enhanced cytotoxicity compared to wild type strains [40]. On the contrary, deletions of the N-terminal have shown reduced seeding abilities in vitro [41]. Post-translational modifications, such as phosphorylation, ubiquitination, acetylation, sumoylation, glycation, glycosylation, nitration and oxidation may also alter the structure, and thereof the function of α-Syn [42, 43].

Likewise, mutations in the gene coding for MAPT may affect the aggregation propensity of Tau by altering its “paperclip” structure, by stabilizing its aggregation core or by influencing the occurrence of PTMs (for a complete review, please see [44]). Moreover, these mutations may induce or prevent alternative splicing events, thus altering the ratio between 3R and 4R isoforms. In a very interesting paper, Strang and colleagues compared the effect of 15 different MAPT mutations on the aggregation properties of Tau upon treatment of cells with wild type K18 Tau (a truncated form of Tau commonly used to seed aggregation) [45]. In this paper, the authors found that the P301L, P301S, and S320F mutations led to a higher degree of aggregation in HEK293T cells [45]. Interestingly, in another comparative study, two mutants, namely L315R and K369I underwent lower aggregation in vitro upon arachidonic acid-induced polymerization [46]. Moreover, in this study, electron microscopy and right-angle laser light scattering analyses showed that different mutations were able to influence the ability of Tau to stabilize microtubules [46]. Notably, strains which differ for their conformational structures may induce the accumulation of specific variants, thus explaining, at least in part, the clinical variability observed in patients [38, 47, 48]. In the case of synucleinopathies, fibril strains with distinct characteristics have been isolated from patients affected by different pathologies, such as PD, MSA or DLB [49]. Similarly, fibrils with different structures have been shown to characterize the brains of patients affected by distinct tauopathies, including AD, PSP and Pick’s Disease [50, 51]. This is important also from a diagnostic point of view, since amplification protocols (e.g. protein misfolding cyclic amplification) associated with biochemical techniques, spectroscopy and cryo-electron tomography can discriminate patients affected by pathologies with partial clinical overlap (e.g PD vs MSA) through the analyses of cerebrospinal fluid (CSF) samples [52]. Finally, aggregates of different natures coexist in the same patient, a phenomenon that suggests an ability of fibrils to cross-seed and interact, likely increasing the level of clinical variability (reviewed in [1, 53]).

Self-propagating seeds are thought to behave as prion-like proteins, alluding to their ability to induce strain/conformational-specific aggregates and subsequently travel to neighboring cells [54]. In cells, this prion-like behavior has been extensively validated [48, 55–57]. In mice, the inoculation of fibrils obtained from pathological human or mouse brain extracts induces protein aggregation in the receiving host [58]. Likewise, newly formed aggregates of α-Syn have been found in human fetal cells years after their transplantation in the brain of PD patients [59].

The prion-like hypothesis pairs up with the Braak staging model, according to which aggregates propagate by following a hierarchical distribution and define a topographical pattern specific for each disease [60]. Although not utterly accepted by the scientific community yet, the Braak hypothesis is constantly growing confidence in the field. This stereotypical march between anatomically related brain regions most likely occurs on account of physical connections held by neighboring cells, which sustains the idea of a non-cell autonomous mechanism at the base of the disease spreading [61]. In support of this, spatial patterns of Tau distribution through synaptically connected neurons have been recently identified in studies analyzing Tau-Positron emission tomography signals in living subjects and integrated with simulating models [62, 63].

## Origins of α-Syn and tau in astrocytes

Both α-Syn and Tau are typically assumed to be neuronal proteins. α-Syn is preferentially located at the pre-synaptic terminals where it is involved in the mobilization of the synaptic vesicles to the pre-synaptic plasma membrane [64]. Tau is a microtubule-associated protein involved in the assembly and maintenance of the microtubule network, which is crucial for the transport of molecules within neurons [65]. Therefore, the discovery of α-Syn and Tau aggregates within astrocytes in patients affected by proteinopathies, has opened new questions about the origin of these astrocytic deposits.

To date, there is little evidence regarding the expression levels and the physiological role of α-Syn in astrocytes, but one hypothesis is that α-Syn may be implicated in astrocytic fatty acid metabolism [66]. Indeed, the neurotoxic effects of α-Syn could be decreased by a reduced fatty acid saturation in neural cells and primary neurons [67]. However, how α-Syn affects fatty acid metabolism in astrocytes should be further investigated. Although present at very low levels, endogenous α-Syn mRNA and protein can be enhanced in vitro by various inflammatory cytokines (e.g. IL-1β) or by conditions of cellular stress in cultured human and rat astrocytes [68, 69]. Astrocytes derived from human induced pluripotent stem cells (iPSCs) obtained from PD patients, express more α-Syn compared to astrocytes derived from healthy patients. This α-Syn is subsequently transferred to healthy ventral midbrain dopaminergic neurons when grown in co-cultures, finally leading to neuronal degeneration [70]. Likewise, Sonninen and colleagues have shown that iPSCs-derived astrocytes obtained from patients with familial PD present an increased amount of α-Syn compared to iPSCs-derived astrocytes obtained from healthy controls, both at mRNA and protein level [71]. In particular, the transcript for SCNA increased along with the differentiation process; interestingly, while PD-derived astrocytes showed more intracellular α-Syn than control-derived astrocytes, there was no difference in the amount of released protein, as shown by ELISA quantification [71]. In contrast, Tsunemi and colleagues have shown that lysates of (healthy) human iPSCs-derived astrocytes do not present endogenous α-Syn inclusions unless when they are co-cultured with dopaminergic neurons derived from iPSCs of patients with SCNA triplication, thus suggesting a neuronal origin for astrocytic α-Syn inclusions [72]. In line with this, transgenic mice expressing α-Syn under a neuronal promoter, such as platelet-derived growth factor B-chain, present α-Syn aggregates both within astrocytes and neurons. Importantly, though, in situ hybridization analyses in these mice show that SCNA mRNA is present in neurons but not in astrocytes, once more suggesting that neurons may be the source of astrocytic α-Syn [73]. The origin of Tau in astrocytes is also controversial since it is commonly believed that astrocytes do not express endogenous Tau. However, some studies suggest that astrocytes do possess an inherent potential to transcribe MAPT. Indeed, a low level of astrocytic MAPT mRNA has been validated in healthy mice and in humans, and its expression may become more pronounced in a pathological context such as glioblastoma [74–76]. However, astrocytes derived from mice injected with AAV ubiquitously expressing either wild type human Tau or a pro-aggregating form of the same (both leading to neuronal tauopathy) did not show an upregulation of the endogenous MAPT gene, as quantified by RT-qPCR [77]. Moreover, despite the low level of Tau expressed by astrocytes in physiological conditions, a study has recently shown that astrocytes obtained from Tau^−/−^ mice present a neuroprotective phenotype in vitro compared to astrocytes obtained from wild type mice, thus suggesting that astrocytic Tau may actually imply a functional consequence [78].

Recently, by using RNAscope imaging and single-nuclear RNAseq, Forrest and colleagues have shown the presence of the MAPT transcript in astrocytes (other than neurons and oligodendrocytes) both in patients affected by PSP and in control subjects [79]. Interestingly, the amount of MAPT in PSP patients was similar between healthy and tufted astrocytes [79]. Moreover, by using the same techniques, Fiock and colleagues found that the number of astrocytes expressing MAPT and the amount of MAPT expressed by each astrocyte is comparable between patients affected by AD, CBD, PSP and healthy controls [80]. Also in this case, the authors did not find differences in the expression of MAPT between astrocytes with or without Tau aggregates in PSP patients [80]. As proposed in the first paper, it is likely that astrocytes express a basal amount of Tau, whose accumulation exacerbates upon the uptake of neuron-released Tau. Alternatively, some still undefined conditions may trigger the autonomous aggregation of Tau within astrocytes [79]. This latter observation could explain why in some pathological conditions, such as PSP, CBD and Pick’s disease, astrocytes show deposits of Tau in the absence of neuronal Tau [81]. However, in contrast with these observations, the knockdown of neuronal Tau expression in transgenic mice inhibits the propagation of Tau pathology in astrocytes, thus suggesting that neuron-derived Tau is necessary for astrocytes to spread the disease [82].

Altogether, these studies suggest that astrocytes may express a very low level of endogenous α-Syn and Tau. A plausible assumption, therefore, is that astrocytes shall inherit pathological protein aggregates from external sources, with neurons being the most likely suppliers.

## Transferring of protein aggregates from neurons to astrocytes

**Fig. 2:**
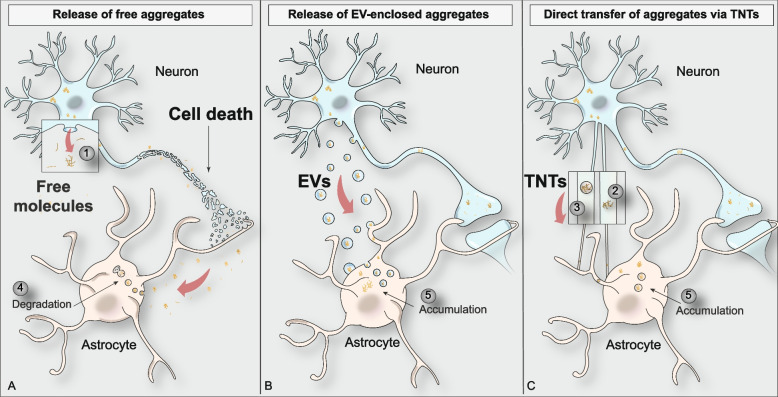
Modalities of amyloidogenic protein transmission from neurons to astrocytes. The figure shows several modalities by which α-Syn and Tau can be transferred from neurons to astrocytes. Specifically: amyloid proteins are released as free molecules by neurons upon degeneration and membrane rupture (1) (**A**). Alternatively, amyloid proteins can be transferred within extracellular vesicles (EVs) (**B**) or by means of Tunneling nanotubes (TNTs), either as free aggregates (2) or within lysosomal-derived vesicles (3) (**C**). Internalized proteins can be partially degraded through the endo-lysosomal pathway (4) or accumulate within astrocytes (5)

The release of proteins lacking a secretory signal peptide (like α-Syn and Tau) generally follows unconventional pathways that do not rely on the involvement of the endoplasmic reticulum and of the Golgi apparatus. The secretion of these proteins may instead occur through the formation of a pore in the plasma membrane or through the release of membranous organelles that fuse with the plasmalemma [83]. Alternatively, proteins can be released in the extracellular environment upon cell damage and death [83, 84] (Fig. 2A). Secreted proteins can therefore be found as free molecules or enclosed within vesicles. In agreement, α-Syn and Tau are found mainly in their naked form in cell culture-derived media or in human biofluids, but they can also be wrapped within intraluminal vesicles such as exosomes and ectosomes where they maintain their seeding potential [85–87] (Fig. 2B). Indeed, amyloid proteins restrained within exosomes can leak out the vesicles upon endolysosomal permeabilization and work as new seeds [88]. Oligomeric species of α-Syn enclosed within exosomes are internalized by human H4 neuroglioma cells and induce a higher level of apoptosis compared to free α-Syn [89]. In agreement, several reports have shown that α-Syn-containing exosomes isolated from the CSF of patients affected by PD or by dementia with Lewy bodies can induce the oligomerization of α-Syn in vitro and in animal models, thus reinforcing the idea that exosomes may efficiently contribute to the spreading of the pathology [90].

Similarly, Tau is retained within the intraluminal side of exosomes, and the release of Tau-containing exosomes has been shown to occur in neuroblastoma cells, primary cortical neurons and organotypic cultures. Interestingly, even though exosomal Tau represents a minimal fraction of total extracellular Tau, exosomes derived from the condition media obtained from cultured neuroblastoma cells (N2a), or from the CSF of early AD patients contain significantly higher levels of phosphorylated (but not total) Tau compared to age-matched healthy subjects, although some controversies exist [91]. Tau-containing exosomes derived from the brain of rTg4510 mice (that express the human 4R0N Tau with the P301L mutation) contain a higher amount of phosphorylated Tau compared to control mice and this exosome-derived Tau is able to induce aggregation in acceptor cells [92]. Nevertheless, by using specific antibodies able to recognize phosphorylated Tau, Wang and colleagues showed that exosomal Tau released by rat primary neurons in culture is hypophosphorylated compared to intracellular Tau [93]. Of note, treatment of N2a cells overexpressing a pro-aggregation form of Tau (TauRDΔK) with exosomes isolated from human CSF induces aggregation of Tau, although there is no significant difference in the seeding properties between AD- and healthy subject-derived exosomes [93].

However, although some indications show that astrocytes can uptake α-Syn-containing exosomes released by neurons and upregulate the expression of pro-inflammatory cytokines, these cells show limited ability to receive exosomes compared to neurons and microglia [94]. Therefore, it is likely that this may not represent the major pathway for α-Syn and Tau entry in astrocytes.

Most cells, including neurons and glia, can interact and exchange intracellular material by stretching networks of TNTs, actin-based extensions of the cells (50–200 nm in diameter) which are formed de novo to enable distal contact with neighboring cells [95]. Initially identified as unbranched channels conveying membranes of the endo-lysosomal system, TNTs have been subsequently shown to mediate both electrical and functional coupling by establishing a continuous connection between remote cells [96, 97]. The cargo transported by TNTs is quite heterogeneous and include organelles, cellular vesicles, cytoplasmic molecules and pathogens [98]. TNTs have been suggested to favor the spreading of prion and prion-like proteins, including α-Syn and Tau [99–102] (Fig. 2C). Of relevance, Abounit and colleagues have shown that the treatment with α-Syn fibrils induces the formation of TNTs in catecholaminergic mouse neuron-like cells (CAD cells) and in primary neurons [101]. In particular, the authors showed that α-Syn fibrils travel within TNTs enclosed in lysosomal-derived vesicles, thus favoring the transfer of α-Syn from donor to acceptor cells [101]. TNT-based intercellular exchange of α-Syn occurs also in SH-SY5Y cells, in primary human brain pericytes and between astrocytes derived from human embryonic stem cells [103, 104]. Interestingly, healthy astrocytes were shown to use TNTs to transfer functional mitochondria to α-Syn exposed astrocytes, thus suggesting that these structures may serve as a rescue strategy [104].

Similarly to α-Syn, overexpressed Tau (2N4R and 1N4R) was found to favor the formation of TNTs and to be transported within these structures between communicating CAD cells as well as between primary neurons [100, 105]. TNTs-mediated transport of Tau (and β-amyloid) has been shown also in glial cell lines in stress conditions that favor the formation of TNTs [106]. Fibrils composed of a truncated form of Tau (K18) as well as Tau obtained from AD brain extracts can propagate between CAD or SH-SY5Y cells within TNTs and are able to seed the formation of new aggregates upon intercellular transfer. Similar results have been observed also in organotypic cultures where Tau aggregates can be received by both neurons and astrocytes through TNT-mediated transfer [107].

### Astrocyte-mediated phagocytosis of aggregated proteins

**Fig. 3:**
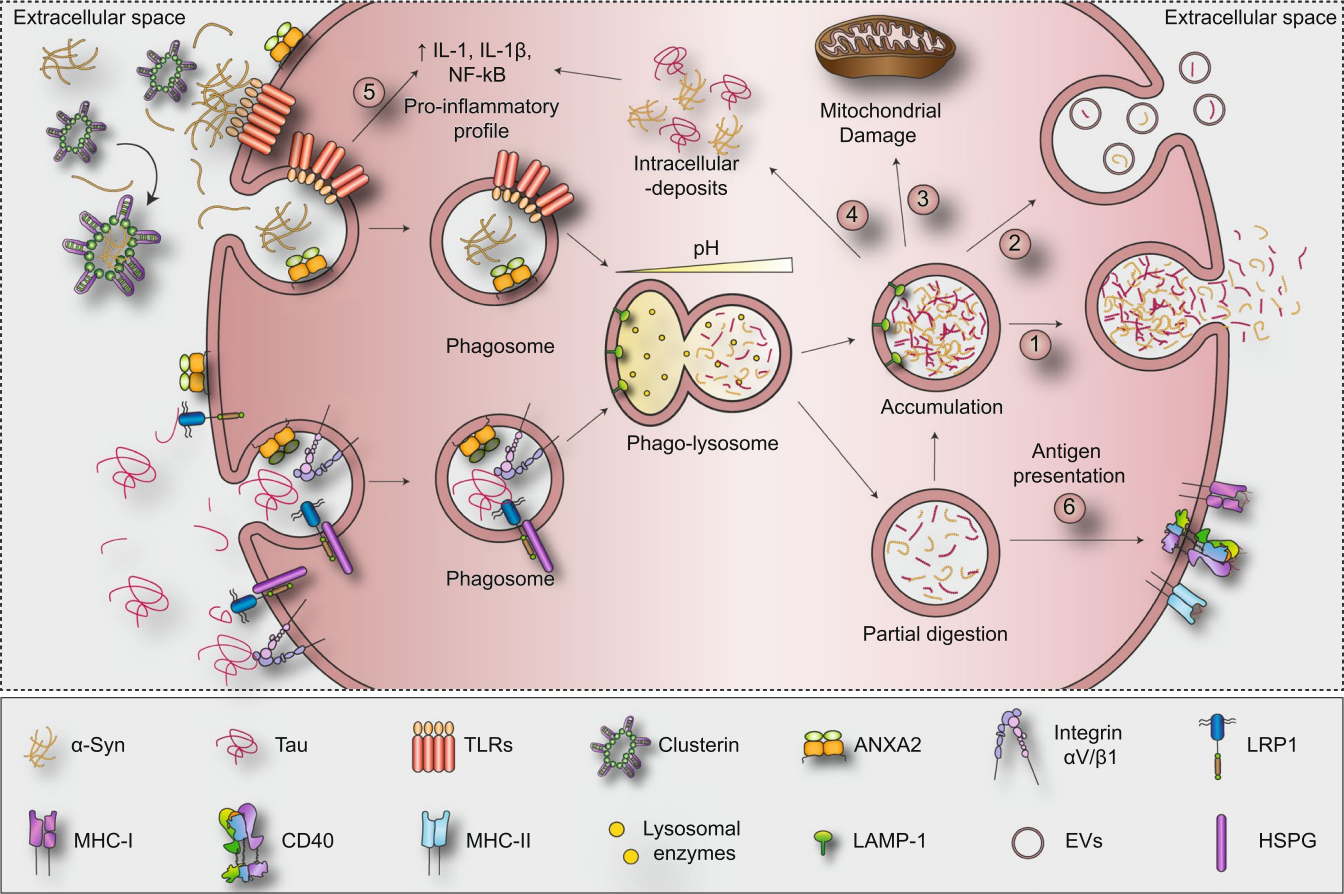
Receptor-mediated phagocytosis of amyloidogenic proteins and processing in astrocytes. The figure recapitulates the different receptors able to interact with α-Syn and Tau, as described in the text. Once recognized by the appropriate receptor(s), α-Syn and Tau are enclosed into phagosomes, which fuse with lysosomes into phago-lysosomes, and subsequently degraded. However, likely due to a high pH into the phagolysosomes, astrocytes cannot perform a complete digestion of the phagocytosed material, which can then be accumulated and released in the extracellular space as free molecules (1) or within vesicles (2). Moreover, the progressive accumulation of α-Syn and Tau can lead to mitochondrial damage (3) or to the formation of deposits (4) that, together with the activation of TLRs (5) can induce a pro-inflammatory phenotype of the astrocytes. Furthermore, astrocytes can expose partially digested antigens on MHC-I, MHC-II or CD40 molecules (6), thus working as antigen presenting cells

Astrocytes can collect extracellular aggregates by means of receptor-mediated phagocytosis/pinocytosis (Fig. 3). Toll-like receptors (TLRs) are among the most promising candidates for the uptake of α-Syn in astrocytes. TLRs are part of the pattern recognition receptors family, and their overexpression in both neurons and glia have been documented both in PD patients and in transgenic mice overexpressing α-Syn [108, 109]. Treatment of astrocytes with conditioned media obtained from SH-SY5Y cells overexpressing α-Syn induces the upregulation of TLR2 and their switch to a pro-inflammatory gene expression profile [73]. In accordance, the administration of anti TLR2 antibody in mice expressing α-Syn under the Thy1 promoter reduces the proinflammatory profile of astrocytes, most likely due to a decreased transfer of α-Syn from neurons to astrocytes [109]. Of note, TLRs are expressed also by microglia, and, similarly to astrocytes the treatment of rat microglia with α-Syn induces the switch to a pro-inflammatory phenotype through the activation of TLR2 [110]. Likewise, the activation of TLR4 mediated by different forms of α-Syn (soluble, fibrillary and truncated) promotes the phagocytic activity in microglia and its transition to an activated status, which is prevented in microglia derived from TLR4^−/−^ mice. Similarly, TLR4 activation does determine a pro-inflammatory status in astrocytes but the presence of the receptor in these cells is not necessary for the uptake of α-Syn [111].

However, a recent study reported that a member of the low-density lipoprotein receptor (LDLR) family, LRP1, that is expressed both in microglia and astrocytes (see below), mediates the internalization of aggregated proteins including Tau and α-Syn in neurons [112]. Notably, the expression of LRP1 together with the activation of TLR4, could reduce the proinflammatory response in macrophages in vitro [113, 114]. Therefore, it would be interesting to investigate whether the expression of LRP1 may play a critical role in the pro-inflammatory profile shift through the activation of TRL4 and others TRLs receptors in astrocytes.

Several reports have been investigating the presence of alternative mechanisms adopted by astrocytes to internalize α-Syn (Fig. 3). Filippini and colleagues recently proposed a role for clusterin, an extracellular chaperon protein which interacts with α-Syn fibrils (but not with monomers) and affects their conformation and aggregation state, finally reducing their internalization [115]. Indeed, clusterin KO primary murine astrocytes and clusterin knock-down human iPSCs-derived astrocytes, internalize a higher amount of extracellular α-Syn fibrils [115]. Considering that clusterin is a ligand for low-density lipoprotein receptor-related protein 8 (LRP8/ApoEr2), a receptor expressed in astrocytes, LRP8 may be involved in this mechanism, even though there is no data regarding its involvement in astrocyte-mediated α-Syn internalization [116].

Our group has recently proposed AnnexinA2 (ANXA2) as a novel modulator of α-Syn uptake in astrocytes [117]. ANXA2 is an actin-binding protein involved in the regulation of intracellular trafficking; in particular, ANXA2 regulates the phago/endocytic pathway by providing stability to endosomes and the abolishment of its expression leads to dysfunctions in the endo/phagocytic system [118]. Of note, ANXA2 interacts with Tau, thus suggesting a possible role in the regulation of Tau/microtubule interaction. In accordance, ANXA2 is upregulated in astrocytes surrounding senile plaques and degenerating neurons in patients affected by AD and ANXA2 has been found to be one of the major components of synaptosomes obtained from AD patients and from mice overexpressing the P301S pathological form of Tau [119, 120]. We have shown that the downregulation of ANXA2 in primary striatal astrocytes reduces the clearance of extracellular small aggregates of human recombinant α-Syn fibrils. In our setting, ANXA2 expression correlates with the activity of Leucine-rich repeat kinase 2, a kinase involved in the pathogenesis of both familial and sporadic PD, thus suggesting a possible contribution of ANXA2 in the pathogenesis of PD [117].

In a proteomic study where rat neurons and astrocytes were treated for a short time (10 minutes) with oligomeric or fibrillar forms of α-Syn, several candidate interactors were identified. The authors suggested that the specific involvement of these interactors could be dependent both on the conformation of the proteins and on the nature of the receiving cell (astrocytes vs neurons) [121]. These observations are in accordance with further studies investigating the role of the heparan sulfate proteoglycan (HSPG) in the uptake of amyloidogenic proteins. Indeed, HSPG participates in the internalization of extracellular α-Syn fibrils, but not oligomers, in both neurons and oligodendrocytes, but it does not seem to be involved in microglia- and astrocyte-mediated recognition of α-Syn in vitro [122]. Strikingly, HSPG is engaged in the macropinocytosis of Tau in neurons, possibly facilitated by the presence of several heparin-binding domains within Tau structure [123]. Indeed, HSPG allowed the binding and the internalization of Tau (both the RD domain and the full length 2N4R isoform) in C17.2 cells. Moreover, primary hippocampal neurons do not uptake Tau fibrils in the absence of Ext1, a protein involved in the synthesis of HSPGs [123]. In the same paper, the authors showed that the injection of a heparin mimetic in mice cortex determines a reduction in neuronal uptake of Tau [123]. Interestingly, treatment of primary astrocytes with heparin or heparinase (to compete with or abolish the functionality of HSPG) does not impair astrocyte-mediated uptake of Tau, thus suggesting that HSPG may not be as crucial as in neurons [124, 125]. However, HSPG mediates the internalization of the monomeric 0N4R Tau splice variant in glioma cells, once more reinforcing the idea that the mechanisms used for the uptake of Tau (and maybe α-Syn) in astrocytes may differ according to the conformational properties of the amyloid protein [126]. Also, the molecular architecture and the nature of PTMs of HSPG itself may influence its interaction with binding proteins [127]. HSPG can act in concert with other receptors, such as LRP1, which is implicated in Tau internalization and spreading. Indeed, the knockdown of LRP1 reduces the uptake of both Tau monomers and oligomers in glioma cell lines and its expression depends on the presence of HSPG [126, 128]. Therefore, although HSPG is not crucial for the internalization of α-Syn and Tau in astrocytes, its presence may be necessary to allow LRP1-mediated uptake.

Intriguingly, both HSPG and LRP1 have been implicated also in the internalization of Aβ in hypothalamic neurons and in N2a cells. Moreover, neuronal-specific downregulation of LRP1 in a mouse model of AD (the APP/PS1 mouse model) crossed with mice expressing either human Apolipoprotein E3 (APOE3) or APOE4 showed that LRP1 is crucial for APOE4-mediated Aβ pathology, thus suggesting that multiple pathways cooperate to internalize extracellular aggregates of different nature [129, 130]. Finally, by using mass spectrometry analysis, Wang and Ye identified αV/β1 integrin as a novel receptor able to promote the internalization of both Tau monomers and fibrils in a HSPG independent way [131].

As already mentioned, many of the proteins involved in the phagocytic process are expressed also by microglia, with which astrocytes embark on a collaborative bi-directional crosstalk for the removal of supernumerary synapses and dead cells [14, 132]. Microglia-depleted mice may therefore be very useful to isolate the role of these receptors in astrocytes and their specific contribution to the clearance of amyloid aggregates.

### Astrocyte-mediated phagocytosis as a mechanosensitive process

Mechanotransduction should also be considered as a possible contributor of aggregates internalization mediated by astrocytes [133–135]. As a matter of fact, physical constraints may define the phagocytic process and a delicate balance between all forces is needed. In this context, the role of the multiple players - i.e. composition and stiffness of the extracellular matrix (ECM), plasma membrane lipid composition and availability - have been widely characterized in professional phagocytes [133–135]. Indeed, during phagocytosis, both the plasma membrane and the cellular cortex need to be significantly reorganized and the ability of the cell to reorganize itself highly depends on the extracellular environment. In particular, the coordination between the cell and the ECM is essential for the interaction of the cell with its target. The lack of adhesion sites between the cell and the ECM might hinder the pickup of the phagocytic target, and it can rather jostle it away [133]. Moreover, the phagocytic efficacy and kinetics also depend on the target shape, size and rigidity [136]. For example, the target shape and rigidity regulate the ability of the cell to organize its actin filaments. Studies on macrophage-mediated phagocytosis have shown that upon the engagement of the target, the cell pushes the particles away for a few micrometers and rapidly pull it back, facilitating in this way the target ingestion [136]. Moreover, it has also been demonstrated that macrophages rotate the rod-shaped substrates in a stiffness dependent manner, until the long axis is pointed toward the cell, to facilitate their pickup [136]. The ability of the phagocytic cells to recognize the physical properties of the target might depend on mechanosensitive regulators. A recent study has demonstrated a role of the mechanosensory channel Piezo1 in the regulation of microglial-mediated phagocytosis of Aβ plaques [137, 138]. In particular, activation of Piezo1 by Aβ plaques induces a calcium influx, which, in turn, causes microglial clustering and phagocytosis [137]. The contribution of mechanosensitive modulators to astrocytic phagocytosis, especially in the context of extracellular aggregates, is currently unknown, but future studies in this direction may open a new perspective to define their phagocytic properties and to plan novel pharmacological interventions.

### Elimination of protein aggregates by astrocytes

Initial reports with in vitro neuron-astrocytes co-cultures and organotypic-based studies have shown that astrocytes degrade intracellular aggregates of α-Syn more efficiently than neurons [72, 139]. Importantly, these studies show that the degraded material is not discharged in the medium and the translocation of α-Syn from astrocytes to neurons is very limited, thus suggesting that the overall effect of astrocyte-mediated clearance is be beneficial, and that astrocytes do not contribute to the propagation of the disease [139]. However, results coming from Erlandsson lab have shown that upon extensive uptake in vitro, astrocytes exhibit large intracellular deposits, most likely due to the overwhelming activation of the endo-lysosomal machinery [102, 140]. This leads to an incomplete digestion, which is indicative of an impaired phagocytic activity. Indeed, astrocytes do not completely degrade the engulfed material, which is therefore accumulates and induces mitochondrial damage. This impairs the astrocytes’ capability to support neuronal metabolism and synaptic function [102, 140]. For example, in human primary astrocytes, excessive α-Syn accumulates into the mitochondria causing reduced oxygen consumption and reactive oxygen species generation [141]. Consistent with this evidence, postmortem analyses showed loss of complex I activity and oxidative damage in the brain of sporadic PD patients [142]. Similar results have been obtained upon Aβ exposure, whose prolonged intracellular storage causes severe endosomal/lysosomal defects in astrocytes, which finally release Aβ-containing vesicles in the surrounding extracellular space [143]. Notably, the acidity of lysosomes is reduced by the activity of Rab27a, which is highly expressed by astrocytes [144]. Indeed, Rab27 recruits Nox2, which prevents the acidification of phagosomes, thus influencing the alkalinization of astrocytic lysosomes that cannot reach the ideal pH to promote full degradation [144, 145]. Nevertheless, the recruitment of Nox2 has been described only in the degradation of dead cells in vitro, hence its role in aggregated protein clearance needs to be further explored [144]. According to a recent hypothesis, the prolonged stagnation of extracellular material into lysosomes may be intentional, since astrocytes could work as antigen presenting cells (APCs), where the degradation of the ingested material has to be slow, in order to obtain smaller fragments to be presented to T-helper cells [146]. In accordance with this hypothesis, healthy astrocytes derived from hiPSCs overexpress the major histocompatibility complex II (MHC-II) upon treatment with α-Syn fibrils. Moreover, brain sections obtained from PD patients and healthy controls show that almost 50% of MHC-II positive cells are indeed astrocytes [146, 147]. In addition, astrocytes upregulate the expression of APCs-related molecules such as CD40, CD80 and CD86 upon stimulation with sonicated fibrils of α-Syn [146]. Reactive astrocytes expressing CD80 and CD86 have also been identified in patients affected by multiple sclerosis, once more suggesting their potential to work as APCs [148]. Nevertheless, several controversies exist, since reports have shown that human fetal astrocytes express little CD80 or CD86 mRNA and protein both in unstimulated and cytokine-stimulated conditions [149, 150]. Moreover, the in vivo expression of APCs-related molecules needs to be further validated. Interestingly, human microglial expression of APCs-related molecules (MHC-I, MHC-II, CD80, CD86, CD40) does not change upon treatment with α-Syn fibrils in vitro [146]. However, it has also been shown that a small population of microglia increase their expression of MHC-II in mice upon the injection of Aβ-Th1 cells, actually working as APCs and contributing to the clearance of Aβ-pathology [151]. However, it is important to point out that most of these studies have been conducted in vitro, a setting that only partially recapitulates the authentic microglia-astrocyte interplay occurring in vivo.

Some studies suggest that upon internalization, also Tau is processed along the major degradative pathways in astrocytes [124, 152]. Indeed, compared to naïve cells, mouse astrocytes overexpressing the transcription factor EB - a master regulator of autophagy-lysosomal biogenesis [153] - phagocytose a higher amount of Tau fibrils, which then co-localize with the lysosomal-associated membrane protein 1, a protein involved in autophagic and endo-lysosomal protein degradation [124]. In accordance, iPSCs-derived astrocytes obtained from patients affected by sporadic or familial AD can engulf both monomeric Tau 2N4R and aggregated Tau, which is thereafter confined to the lysosomal compartment [152].

The progressive accumulation of protein aggregates may affect astrocytes and impair their overall functionality (Fig. 3). Excessive inclusions in astrocytes upon exposure to α-Syn preformed fibrils in vitro induce the release of multiple pro-inflammatory cytokines (e.g. IL-1α, IL-1β) and chemokines (e.g. CC- and CXCL-type), increase their neurotoxicity and impair their phagocytic ability [73]. Dysfunctions in astrocytes may also translate into loss of neuroprotective properties. Astrocytes derived from mice overexpressing the P301S pathological form of Tau under the control of the neuronal Thy1.2 promoter show reduced ability to support degenerating neurons [154], while the selective expression of A53T α-Syn in astrocytes induces astrogliosis and disrupts normal astroglial function leading to neurodegeneration in mice [155]. IPSC-derived astrocytes obtained from both familial and sporadic PD patients co-cultured with midbrain dopaminergic neurons induce morphological changes indicative of neuronal degeneration [70]. Although this has been attributed to the secretion and transfer of α-Syn from astrocytes to neurons, the release of additional toxic molecules that affect neuronal survival could not be ruled out [70]. On the contrary, however, astrocytes derived from iPSCs obtained from AD patients exert beneficial effects on Tau-exposed iPSCs-derived neurons by releasing matrix metalloproteinases (MMPs) [152]. Exposure of cultured mouse astrocytes to Tau oligomers significantly affects the frequency and amplitude of Ca^2+^ transients and the consequent release of gliotransmitters, ultimately resulting in synaptotoxic effects [156]. In particular, the presence of extracellular oligomeric Tau alters the functionality of the astrocytic Na^+^/K^+^ ATPase (NKA) while its internalization impairs the activity of the excitatory amino acid transporters, whose proper expression is crucial in both PD patients and in PD experimental animal models [157, 158]. Interestingly, also α-Syn interacts with the neuronal α3-subunit of NKA, causing its clustering and relocalization, finally impairing the maintenance of Na^+^ gradient [159]. Moreover, Tau internalization has been associated with morphological alterations and enhanced reactivity of astrocytes. Exposure to monomeric or fibrillary Tau induces the activation of the extracellular signal-regulated kinases ERK1/2 and the expression of cytokines and chemokines such IL-6 and tumor necrosis factor-α (TNF-α), switching astrocytes to a pro-inflammatory phenotype [126].

Altogether, these studies suggest that astrocytes can internalize extracellular protein aggregates, but their degradative potential is quite limited. As a consequence, significant doubts remain regarding the overall outcome of astrocyte-mediated clearance in proteinopathies.

## Targeting glial cells to block proteinopathies

**Fig. 4:**
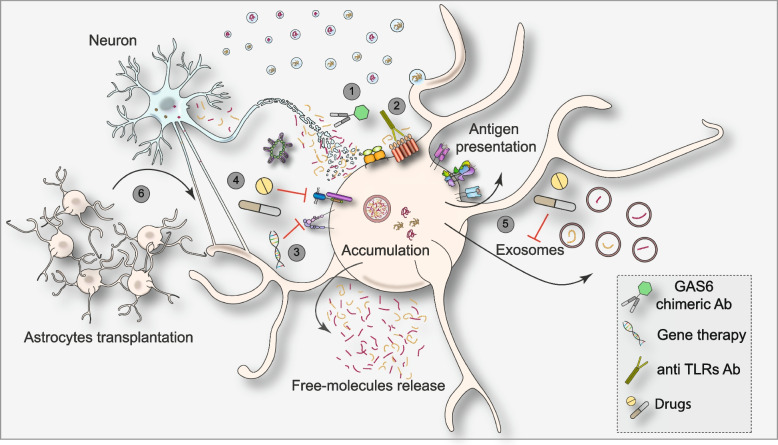
Possible astrocyte-focused strategies to treat proteinopathies. The figure recapitulates the modalities of α-Syn and Tau transmission from neurons to astrocytes, as well as the possible fate of the ingested proteins. Moreover, possible strategies to treat proteinopathies are highlighted, such as the use of monoclonal antibodies fused with GAS6 to specifically direct phagocytosis (1) the application of anti-TLRs antibodies (2) and gene therapy/gene editing (3) to regulate the internalization of amyloid proteins; the use of drugs to modulate the interaction of amyloid proteins with receptors like HSPG or LRP-1 (4) or to reduce the release of toxic protein within exosomes (5). Moreover, the transplantation of patients’ derived astrocytes may represent an alternative therapeutic approach to favor extracellular clearance (6)

The growing awareness of astrocytes’ contribution to both the physiology and pathology of the CNS has led scientists to consider these cells as potential targets for the development of novel therapeutic strategies (Fig. 4). In the past years, great attention has been addressed to active immunization as a potential approach to treat proteinopathies, and several vaccines against Aβ have been raised, some of which are currently being tested in clinical trials [160]. In a recent study, Jung and colleagues engineered a novel antibody to induce microglia and astrocyte-mediated phagocytosis while reducing secondary inflammation, often associated with antibody-based immunotherapies [161]. Specifically, the authors took advantage of the molecular structure and biological function of the Growth Arrest Specific 6 (GAS6) protein. GAS6 binds both phosphatidylserine and TAM receptors thanks to its N-terminal and laminin G-like domains, respectively, thus bridging dying cells (exposing phosphatidylserine) to microglia and astrocytes. By replacing the N-terminal domain of GAS6 with the single-chain fragment variable of a validated Aβ-targeting monoclonal antibody, the authors obtained a molecule able to induce microglia and astrocyte-mediated phagocytosis of extracellular Aβ, upon infusion in models of AD [161]. Therefore, in the future, a similar approach could be used to target α-Syn or Tau aggregates. Alternative approaches directed to the elimination of amyloid protein aggregates based on oligonucleotide therapies, gene therapies and gene editing as well as targeted protein degradation and stem cell therapies are also being developed (for a recent review, see [160]).

So far, however, only a few studies have investigated the application of therapies to modulate astrocytic clearance of amyloid proteins, most likely due to the poor knowledge of the molecular machinery involved. As mentioned above, TLRs are implicated in the internalization of α-Syn; therefore, the application of molecules able to target these receptors may represent a valid pharmacological strategy for proteinopathies, although most of these drugs have mainly been tested in patients affected by cancer [162]. A study in a preclinical model of synucleinopathies has shown that the administration of anti-TLR2 improves the neuropathology and behavioral outcome by limiting neurons-to-astrocytes transmission of α-Syn, thus boosting new hopes for future clinical applications of TLR2 antagonists in the field of PD [109]. Likewise, targeting of HSPG and/or its interaction with LRP1 may reduce Tau internalization. For instance, molecules that interfere with HSPG expression may be worth to be studied, since they can indirectly affect the activity of other receptors, such as LRP1. So far, molecules able to antagonize HSPG activity and/or expression, including monoclonal antibodies, antibiotics, and peptides, are currently being tested mainly in cases of cancer [163]. Future strategies including pharmacological and gene therapy treatment to modulate the expression of LRP-1 may also be considered. Molecules able to induce LRP1 expression, such as Rosiglitazone, which has shown beneficial effects in APOE ε4-negative patients (but not in APOE ε4-positive patients) may be re-evaluated in future studies [164]. However, we should consider that receptors such as TLRs and LRP1 are expressed also by microglia, other than astrocytes. Therefore, any protocol aimed at modulating the functionality/interactions of these receptors might have effects on the microglial population as well. Compelling evidence has shown that starvation induces an increase in ANXA2 expression correlated with an increase in the autophagic pathway [165]. Of note, it is emerging that diet habits might have important implications for brain health, and for several brain disorders as PD [166]. Therefore, the modulation of ANXA2 levels through diet might have beneficial effects on protein aggregates uptake, reducing PD progression. More specific approaches to target ANXA2 have been recently tested. For example, natural compounds such as ginsenosides Rg5 and Rk1, as well as a plant lectin and a plant alkaloid, have been found to interact with ANXA2 and inhibit its downstream pathway in cells [167]. Likewise, DNA/RNA hybrid nanoparticles, shRNA, miRNA and a monoclonal antibody against ANXA2 have shown some pre-clinical effect in cellular and animal models of cancer [167]. However, while these approaches aim at reducing the expression or functionality of ANXA2, no strategies have been investigated, to the best of our knowledge, to upregulate the expression of this protein, an instance that would be desired in the case of proteinopathies.

However, as described, astrocytes are not very efficient at digesting the engulfed material. Therefore, whether the induction of astrocyte-mediated clearance may be beneficial or may aggravate the pathological condition, still needs to be defined. In this scenario, the application of a therapy to induce astrocytic digestion-other than protein internalization- may be advantageous to ensure an efficient clearance of the extracellular environment. A recent study points to astrocytes as potential targets for the proteolysis of aggregates in Huntington’s disease pathology. Indeed, Abjean and colleagues, describe that the activation of JAK-STAT3 pathway in reactive astrocytes could decrease mutant Huntingtin aggregation and increase its degradation, which in the end, helps neurons in handling toxic protein and preventing degeneration [168].

Alternative strategies may focus at reducing the spreading of the pathology. In this context, the calcium-sensing receptor antagonist NPS 2143 significantly reduces the amount of phosphorylated Tau in astrocyte-derived exosomes upon exposure to Aβ_25–35_ in cortical adult human astrocytes [169]. Recently, the transplantation of astrocytes derived from the ventral midbrain region lessens neuronal accumulation and spreading of α-Syn, thus suggesting a novel therapeutic approach [170]. Considering the technological acceleration we have been witnessing in the past few decades and the escalation of knowledge that this brings, we can speculate that in the upcoming years, novel mechanisms lying behind astrocyte-mediated clearance will be revealed, hopefully leading to effective astrocyte-specific therapeutic approaches.

## Conclusions

Due to the growing extension of life-expectancy, the impact of proteinopathies is anticipated to quickly escalate in the next future. Therefore, efforts at finding new therapeutic strategies to slow the progression of these chronic and thus far incurable diseases are highly desirable. While the causal events triggering the onset of these pathological conditions have not been fully disclosed yet, progressive advances have allowed the identification of new cellular and molecular players involved in the later phases of these diseases. It is now recognized that proteinopathies are not pure “neuronal” pathologies, but they do involve a more heterogeneous population of cells, whose functionality becomes affected in a diseased environment. Although the role of astrocytes in proteinopathies is not fully defined yet, growing evidence suggests that the loss of proper astrocyte-mediated clearance activity might contribute to the onset and/or progression of several proteinopathies. As described, proteinopathies are distinguished by a common sequence of aggregates formation and propagation, an aspect that is both provocative and appealing at the same time. Therefore, it is reasonable to wonder whether there are common mechanisms involved in the removal of these aggregates, or if protein-specific mechanisms are instead at play, an aspect that would obviously lead to very different therapeutic strategies. We have discussed here several studies reporting the role of specific receptors in the engulfment of α-Syn and Tau. Nevertheless, results have been contrasting in some cases (e.g. HSPG). It is possible that these discrepancies may be due to the use of proteins with different properties (e.g. monomers vs oligomers). Moreover, both α-Syn and Tau exist in several conformations, characterized by distinct aggregation dynamics. Therefore, it would be important in the future to understand if the internalization of amyloid proteins may depend on the properties of a specific strain, the presence of certain PTMs or on the nature of the protein itself. Overall, it is tempting to think that astrocytes may adopt different strategies to remove distinct aggregated proteins from the CNS. However, as discussed, astrocyte-mediated engulfment of extracellular aggregates may have a deleterious effect, due to their switch to a pro-inflammatory state and their contribution to the disease spreading. In this case, therefore, strategies aimed at blocking the ingestion of extracellular aggregates may instead be desirable. Crucially, astrocytes display anatomical, morphological and molecular heterogeneity, which leads to both regional and local diversity [171, 172]. These structural differences, which have been described both at transcriptomic and proteomic levels, are reflected by distinct physiological and functional properties [173]. Moreover, these differences may be further enhanced in pathological conditions, thus adding an extra level of complexity [174]. Therefore, it is likely that distinct subpopulations of astrocytes may respond differently to a given therapeutic strategy, an observation that needs to be taken into account when approaching pathologies with specific brain region etiology.

Despite many steps forward have been made in the comprehension of the molecules and pathways involved in astrocyte-mediated clearance, multiple questions still remain unresolved. Answering these questions will advance our understanding of astrocytes contribution in proteinopathies and will hopefully lead to the development of novel therapeutic strategies.

### Supplementary Information

**Additional file 1: Suppl. Table 1.** Studies reporting how mutations and PTMs of α-Syn and Tau can affect their conformation and physiological properties.

## Data Availability

Not applicable.

## Abbreviations

*α-Syn*: α-Synuclein
*Aβ*: Amyloid-β
*AD*: Alzheimer Disease
*ANXA2*: Annexin A2
*APCs*: Antigen Presenting Cells
*APOE4*: Apolipoprotein E4
*APRs*: Aggregation prone regions
*CD*: Cluster of differentiation
*CNS*: Central Nervous System
*CSF*: Cerebrospinal fluid
*ECM*: Extracellular Matrix
*GAS6*: Growth Arrest Specific 6
*HSPG*: Heparan Sulfate Proteoglycan
*IPSCs*: Induced Pluripotent Stem Cells
*LDLR*: Low-Density Lipoprotein Receptor
*LRP1*: Low Density Lipoprotein Receptor-Related Protein 1
*LTP*: Long-term potentiation
*MAPT*: Microtubule-Associated Protein Tau
*MEGF10*: Multiple EGF Like Domains 10
*MERTK*: MER Proto-Oncogene Tyrosine Kinase
*MHC*: Major Histocompatibility Complex
*MMPs*: Matrix Metalloproteinases
*NAC*: Non-Amyloid Component
*NDs*: Neurodegenerative Disorders
*NKA*: Na^+^/K^+^ ATPase
*NMDA*: N-methyl-D-aspartate
*PD*: Parkinson’s Disease
*PTMs*: Post Translational Modifications
*TLRs*: Toll-Like Receptors
*TNF-α*: Tumor Necrosis Factor-α
*TNTs*: Tunneling Nanotubes
*UPS*: Ubiquitin–proteasome system
