# Supplementary material for: Brain clearance of protein aggregates: a close-up on astrocytes
Source: Mol Neurodegener. 2024 Jan 16;19:5. doi: 10.1186/s13024-024-00703-1 (PMC10790381; doi:10.1186/s13024-024-00703-1)
Supplement: Supplementary file 1 — Additional file 1: Suppl. Table 1. Studies reporting how mutations and PTMs of α-Syn and Tau can affect their conformation and physiological properties. [file 13024_2024_703_MOESM1_ESM.docx]

| Suppl. Table 1: Studies reporting how mutations and PTMs of α-Syn and Tau can affect their conformation and physiological properties | | | |
| --- | --- | --- | --- |
| Authors Year | **α-Syn** | **Settings** | **Highlights** |
| Conway et al.(1)  1998 | WT, A53T, and A30P mutants of α-Syn | **in vitro:** CD, α-Syn aggregation, AFM, EM | -WT and both A53T and A30P mutants have similar natively unfolded' structures  -the A53T mutation leads to faster fibrillogenesis than WT and A30P mutation |
| Narhi et al.(2)  1999 | WT and A53T, A30P, and A53T + A30P mutants of α-Syn | **in vitro:** α-Syn aggregation, CD, FTIR, AFM, EM | -all the mutants have a faster kinetics of fibrillogenesis (lag phase) compared to WT α-Syn, with A53T being faster than A30P |
| Ostrerova-Golts et al.(3)  2000 | WT, A53T, and A30P mutants of α-Syn | **in vitro**: WB, EM  **in cells:** α-Syn aggregation, toxicity, ThS, ICC | -in the presence of FeCl_2_, the A53T mutation promotes the formation of more aggregates compared to WT or to the A30P mutation  -treatment of cells expressing WT or A30P α-Syn with FeCl2 and with 50 or 500μm dopamine induces the formation of aggregates  -aggregates of α-Syn that form in neurons in response to iron treatment are ubiquinated. |
| Li et al.(4)  2001 | WT, A53T, and A30P mutants of α-Syn | **in vitro:** α-Syn aggregation, light scattering, CD, FTIR Spectra, Small-angle X-ray scattering, TEM | -all three proteins possess almost identical far-UV CD spectra at acidic pH  -both heating and decrease in pH contribute to the reversible  transformation of α-Syn to a partially folded intermediate  - The structure content of α-Syn increases in the following order: WT < A30P < A53T  -the mutants have a greater propensity to aggregate at higher concentrations than the WT α-Syn  -the A53T mutant shows the fastest rate of fibrillogenesis, whereas A30P show slower fibrillogenesis than the WT protein |
| Lee et al.(5)  2002 | Mice expressing either WT, A53T, or A30P mutants of α-Syn | **in vitro:** WB  **ex vivo:** in situ hybridization, IHC  **in vivo:** behavioural analysis | -mice overexpressing the A53T mutation show earlier disease onset and a greater penetrance of the disease phenotype compared to mice expressing WT α-Syn or α-Syn carrying the A30P mutation  -mice overexpressing the A53T mutation show aberrant (regionally specific) accumulations of α-Syn and ubiquitin  -brains of mice overexpressing the A53T mutation present more detergent-insoluble full-length α-Syn |
| Murray et al. (6)  2003 | WT and  truncated recombinant human α-Syn | **in vitro:** α-Syn aggregation,  turbidity analysis, ThT, ANS binding assay, digestion assay, negative staining EM, Immunoelectron Microscopy, CD | -all of the truncated forms proteins aggregate more rapidly than full-length α-Syn  -the truncated forms of α-Syn produce shorter and thinner fibrils compared to full length  -amino acids at 104, 105 and 114, 115 in the carboxy-terminus contrast α-Syn aggregation |
| Zhou et al.(7)  **2004** | Constructs of WT, A53T, and A30P mutants of α-Syn carrying tyrosine to either cysteine or phenylalanine residues at positions Y39, Y125, Y133, Y136 | **in vitro:** WB, α-Syn aggregation  **in cells:** ICC, cell viability, apoptosis, and α-Syn aggregation | -cells expressing either Y39C or Y125C mutants (of both WT, A53T and A30P strains) form cytoplasmic α-Syn aggregates and lead to cell toxicity  -Dopamine treatment increased α-Syn inclusions in Y39C and Y125C transfected cells compared with WT in N27 cells  -in vitro the Y39C mutation increases the dimer/monomer after H_2_O_2_ treatment compared with WT and other mutants |
| Li et al.(8)  2005 | Mice expressing either WT, A53T, or A30P mutants of α-Syn  Constructs of WT, A53T, A30P and various truncated mutants of α-Syn | **in vitro:** WB, Ciphergen Surface-Enhanced Laser Desorption and Ionization (SELDI)-MS Analysis, α-Syn aggregation | -the expression of α-Syn mutants leads to higher relative levels of α-SynΔC  -α-SynΔC aggregates more readily than α-Syn WT and α-SynΔC increases aggregation propensity of α-Syn WT  -PD patients brains present a high proportion of α-SynΔC in the detergent-insoluble α-Syn aggregates than in the soluble fraction |
| Pandey et al.(9)  2006 | WT, A53T, A30P and E46Kand E46KΔG mutants of α-Syn | **in cells:** α-Syn aggregation, EM  **in vitro:** WB | -cells expressing E46K and E46KΔG present more and larger aggregates than cells expressing A53T, WT or A30P α-Syn |
| Meier et al.(10)  2012 | WT and nine lysine to cysteine mutants (K6C, K10C, K12C, K21C, K23C, K32C, K34C, K46C, and K96C) of α-Syn | **in vitro:** CD, α-Syn aggregation, ThT, TEM, dynamic light scattering, dot blot | -K10C-Ub and K23C-Ub mutants display similar levels of fibrils compared to WT but with different kinetics  -K6C-Ub, K12C-Ub, and K21C-Ub mutants display fewer levels of fibrils compared to WT  - K32C-Ub, K34C-Ub, K43C-Ub, and K96C-Ub mutants display a strong inhibition of fibrils formation  -K96-Ub mutant might promote the formation of oligomers |
| Marotta et al.(11)  2012 | WT α-Syn, 68–77 peptide with or without O-GlcNAc modification | **in vitro:** WB, α-Syn aggregation, CD, dynamic light scattering, ThT, TEM | -O-GlcNAc of the peptide composed of residues 68–77 of α-Syn inhibits the seeding potential of the peptide without the PTM. |
| Ghosh et al.(12)  2013 | WT, H50Q, H50A, A53T and E46K mutants of α-Syn | **in vitro:** NMR, CD, ThT, EM  **in cells:** cytotoxicity assay | -both H50Q and H50A show faster fibrillogenesis than WT α-Syn  -the mutants show similar cytotoxicity to WT α-Syn  -the oligomer/monomer ratio is lower for H50Q and H50A than for WT α-Syn  -H50Q α-Syn exhibited a denser population of spherical and pore like structures  - neither H50Q nor H50A alter the major secondary structure under normal physiological conditions or membrane mimicking conditions, but the H50Q mutation may modulate the interactions  between the NAC region and the C-terminus |
| Bousset et al.(13)  2013 | two different polymorphs of WT α-Syn (fibrils and ribbons) | **in vitro**: α-Syn aggregation, TEM, proteolytic digestion assay, Fourier transform infrared spectra, X-ray fibre diffraction, solid-state NMR;  **in cells**: α-Syn aggregation, cytotoxicity assay, FC | -different polymorphs adopt different conformations  -different polymorphs imprint intrinsic architecture to soluble α-Syn molecules both in vitro and in cells  -α-Syn fibrils are more cytotoxic and bind less efficiently to artificial lipid vesicles  compared to ribbons but increase cell permeabilization  -α-Syn fibrils have higher nucleating and amplifying activities in cells as compared with α-syn ribbons and have different efficiencies of propagation |
| Khalaf et al.(14)  2014 | WT and H50Q α-Syn mutant | **in vitro:** α-Syn aggregation, ThT, TEM, Gel Filtration Chromatography/Static Light Scattering, DC, NMR, AFM, phosphorylation assay  **in cells:** phosphorylation assay, cell viability, IF, FACS | -the H50Q mutation does not alter the shape or size of α-Syn, and induces only little perturbation of α-Syn structure in solution  -the H50Q mutation slightly perturbs the interaction of α-Syn with membranes  -the H50Q mutation induces slightly faster α-Syn fibril formation compared to WT and faster rates of β-sheet-rich oligomer formation  -the H50Q mutation does not affect α-Syn phosphorylation at serine or tyrosine residues, nor does it affect PLK2-mediated degradation of α-Syn.  -the H50Q mutation does not alter the cellular distribution but induces higher secretion of α-Syn compared to WT  -the H50Q mutation induces cellular toxicity and mitochondrial fragmentation |
| Fares et al.(15)  2014 | WT, E46K, H50Q and A53T, A30P and G51D α-Syn mutant | **in vitro:** α-Syn aggregation, ThT, TEM, DC, NMR, Multi angle light scattering, WB, ICC  **in cells:** IF, LDH and Sytox green cytotoxicity assays | -the G51D mutation does not significantly affect the structure of α-Syn in aqueous solution  -the G51D mutation determines a slower aggregation of α-Syn and a slower fibrillogenesis  -the G51D mutation decreases the interaction of α-Syn with membranes and toxicity in yeast  -the G51D mutation increases the nuclear localization and phosphorylation of α-Syn  -the G51D mutation increases mitochondrial fragmentation as well as α-Syn secretion, but not cell death compared to WT α-Syn  -G51D mutated α-Syn is present in inclusions in PD patients |
| Ghosh et al.(16)  2014 | WT, A53T, A53E, A53K  and A30P mutants of α-Syn | **in vitro:** α-Syn aggregation, ThT, CD, AFM, SEC, photo-  induced chemical cross-linking of unmodified proteins, dynamic light scattering, heteronuclear single  quantum coherence (HSQC) spectroscopy, surface  plasmon resonance  **in cells:** cytotoxicity assay | -the A53E mutant has slower fibrillogenesis kinetics compared to WT and other mutants of α-Syn  -A30P and A53E α-Syn accumulate oligomers for a longer duration of time  -A53E α-Syn showed slightly less toxicity at a lower concentration compared to WT and other mutants  -A53E has lower affinity toward membrane compared to WT α-Syn in the absence of lipids  -A53E has similar affinity toward membrane compared to WT α-Syn but slower than A53T in the presence of lipids |
| Lazaro et al.(17)  2014 | 9 α-Syn point mutants | **in cells:** Bimolecular Fluorescence Complementation, ThS, stimulated emission depletion super resolution microscopy, bioluminescent protein complementation assay, LDH  **in yeast**: toxicity assay | -A53T induces the strongest rate of oligomerization  -A30P induces the highest number of aggregates in cells  -phosphomutants reduced the formation of aggregates in cells  -A30P/A56P/A76P triple mutant, A30P, A53T and Y125F promote the presence of extracellular oligomers  -E57K and Y125F mutants co-localize with LAMP-1; E35K and E57K mutants induce higher Golgi fragmentation  -E35K mutant induces S129 phosphorylation |
| Rutherford et al.(18)  2014 | WT and H50Q or G51D mutants of α-Syn | **in vitro:** α-Syn aggregation, EM, AFM, WB  **in cells:** ICC, cell viability | -the H50Q mutation increases, while the G51D mutation decreases α-Syn aggregation compared to WT α-Syn  -neither H50Q nor the G51D mutation alter the ultrastructural properties of α-Syn  -the H50Q mutation induces a higher rate of α-Syn aggregation compared to WT and G51D mutated α-Syn  -H50Q and G51D mutants show less protection against H_2_O_2_ compared to WT and higher toxicity in cells treated with MPP+ |
| Xiang et al.(19)  2015 | WT, H50Q and H50R mutants of α-Syn | **in vitro:** MS, WB, SEC, EM, ThT, aSyn aggregation, dot blot  **in cells:** ICC, FC, cytotoxicity, aSyn aggregation, cell viability assay | -H50 is subjected to HNE modification  -H50 favours HNE-induced oligomerization of aSyn  -H50 favours HNE-induced cytotoxicity  -H50 mutations exacerbate nitration-mediated oligomerization  -H50 mutants favour the formation of aSyn fibrils  -the H50Q mutation but not H50R induces the formation of aSyn aggregates in cells, but both mutants induce apoptosis in cells  -H50 mutations increase the cellular susceptibility to oxidative stress. |
| Rutherford and Giasson(20)  2015 | WT and A53E mutant of α-Syn | **in vitro:** α-Syn aggregation, TEM  **in cells:** α-Syn aggregation, ICC, cell viability assay | -the A53E mutation reduces the formation of α-Syn aggregates and fibrils in vitro and in cells  -the A53E mutation leads to thinner fibrils of α-Syn compared to WT  -the A53E mutation significantly increases cell death at the lowest MPP+ concentration compared to WT |
| Marotta et al.(21)  2015 | WT, T72A mutant of α-Syn and gT72 α-Syn (bearing an O-GlcNAc modification at T72) | **in vitro:** α-Syn aggregation, TEM, ThT, CD, dynamic light scattering, SEC, membrane binding assay, phosphorylation, WB  **in cells:** α-Syn aggregation, cell toxicity assay | -the T72A mutation to prevent O-GlcNAcylation leads to a decreased formation of α-Syn aggregates  -O-GlcNAcylation blocks the formation of both fibers and oligomers and promotes the solubility of α-Syn  -O-GlcNAcylation does not change the binding of α-Syn to membranes  -O-GlcNAcylation may reduce phosphorylation of α-Syn  -O-GlcNAcylation reduces cell toxicity  -O-GlcNAcylated α-Syn remains more soluble during the aggregation reaction and is incorporated with reduced efficiency compared to WT α-Syn |
| Mason et al.(22)  2016 | unmodified and acetylated WT and H50Q α-Syn | **in vitro:** electrospray ionization−ion mobility spectrometry−mass spectrometry, ThT | -acetylation weakens the ability of α-Syn to bind Cu^2+^ both for WT and the H50Q mutant  -acetylated α-Syn undergoes reduced conformational changes upon Cu^2+^ binding compared to non-acetylated α-Syn (less prone to aggregation)  -the H50Q mutation increases aggregation propensity compared to WT α-Syn, with a further reduction in lag time in the presence of Cu^2+^, which, however, does not change in presence of acetylation |
| Flagmeier et al.(23)  2016 | WT, A30P, E46K, H50Q, A53T and G51D mutants of α-Syn | **in vitro:** CD, α-Syn aggregation, ThT, AFM, Differential scanning calorimetry | -the G51D mutant has higher affinity for lipid molecules  -A53T and A30P mutants have higher aggregation rate compared to WT, while E46K, H50Q and G51D have lower aggregation rates compared to WT in the presence of lipids  -E46K, H50Q, G51D, and A53T have the highest rates of elongation compared to WT α-Syn  -H50Q and G51D mutants have a very slow secondary nucleation rate |
| Lazaro et al.(24)  2016 | WT and A53E mutant of α-Syn | **in vitro:** α-Syn aggregation, Light scattering spectroscopy, ThT, Congo red binding assay, TEM, Attenuated total reflectance Fourier transform infrared spectroscopy, WB  **in cells:** α-Syn aggregation, Bimolecular Fluorescence Complementation assay, ICC, ThS, Golgi fragmentation, Proteinase K digestion, FC, Measurement of 26S Proteasome Catalytic Activity, cytotoxicity assay | -the A53E mutation reduce the rate of α-Syn aggregation and leads to the formation of oligomers rather than fibrils with disordered/random coil conformation compared to WT α-Syn in vitro  -cells expressing the A53A mutated α-Syn produce less oligomers compared to cells expressing WT α-Syn  -the A53A mutation does not change the formation of inclusions in cells, nor the phosphorylation of α-Syn, but it leads to less compact aggregates compared to cells expressing WT α-Syn  -aggregation of A53E α-Syn induces more Golgi alterations compared to WT α-Syn  -in yeast, the expression of the A53A mutant does not chance cytotoxicity, inclusion formation and/or phosphorylation compared to WT α-Syn |
| Íñigo-Marco et al.(25)  2017 | WT, A30P, E46K, and A53Tmuants of α-Syn | **in vitro:** WB  **in cells:** IF | -the E46K mutation leads to the most toxic pathological α-Syn in cells compared to all the strains analysed  -the toxicity observed with the E46K mutation is not due to an increase in protein stability or aggregation  -the E46K mutation leads to higher PLK2-driven phosphorylation compared to WT α-Syn but does not explain the toxicity of the E46K mutant  -E46K α-Syn toxicity is predominantly cell autonomous |
| Iyer et al.(26)  2017 | C-terminal truncated variant of α-Syn (1–108) | **in vitro**: α-Syn aggregation, ThT, Scanning EM, AFM, CD, 1D- and 2D-IR and VCD Spectroscopy, Steady-State Fluorescence Spectroscopy, X-ray Fiber Diffraction | -α-Syn (1–108) cannot seed with WT α-Syn monomers  -while WT and α-Syn (1–108) monomers have similar spectra, α-Syn (1–108) fibrils show higher twisting than TW fibrils  - α-Syn (1–108) has faster aggregation kinetics |
| Afitska et al.(27)  2017 | WT and C-terminal modified α-Syn (α-Syn-2Asn, α-Syn-5Asn, and α-Syn-9Asn, mutant α-Syn-5Lys and mutant α-Syn-5Asp) | **in vitro:** α-Syn aggregation, ThT, AFM, CD | - primary nucleation rate strongly depends on both the charge and the length of α-Syn  - modification of the α-Syn C-terminal charge does not significantly affect the fibril elongation rates at physiological salt concentration |
| de Oliveira and Silva (28)  2019 | WT, A30P, E46K, and A53T mutants of α-Syn | **in vitro:** TEM, α-Syn aggregation, ThT, digestion assay, CD, cryo-EM, electrospray ionization mass spectrometry | -ThT, digestion assay and cryo-EM analyses show conformation similarities between WT and A53T mutant α-Syn compared to A30P and E46K α-Syn  -In the presence of increasing amounts of seeds, A53T α-Syn increases the fibril mass in the presence of physiological relevant ionic strength  -A53T α-Syn forms amyloid fibrils via secondary nucleation processes at neutral pH under quiescent conditions |
| Boyer et al.(29)  2019 | WT and H50Q (Narrow and Wide Fibrils) mutant of α-Syn | **in vitro**: Negative stain TEM, ThT, SDS stability, Cryo-EM, atomic modelling and energetic calculation;  **in cells**: α-Syn aggregation, mitochondrial activity assay and LDH | - the H50Q mutation results in two polymorphs (Narrow and Wide Fibrils) which differ for the sequence of the conserved kernel used to form protofilaments  -WT and H50Q show a similar resistance to denaturation  -the H50Q mutant has faster aggregation kinetics  -the H50Q mutant has higher seeding capacity in cells and cytotoxicity compared to WT α-Syn |
| Zhao et al.(30)  2020 | N-terminally acetylated WT and E46K mutant of α-Syn (Ac-WT and Ac-E46K) | **in vitro**: CD spectroscopy, denaturation, TEM, AFM, proteolytic digestion assay, ThT, cryo-EM, sonication | -the Ac-E46K mutant is more twisted than Ac-WT α-Syn and show right-handed helical twist  -the Ac-E46K mutant is less table than Ac-WT α-Syn  -the Ac-E46K mutant has higher seeding capacity than Ac-WT α-Syn |
| Doherty et al.(31)  2020 | N-terminal truncated variant of α-Syn lacking residues lacking 36GVLYVGS42 (ΔP1), 45KEGVVHGVATVAE57 (ΔP2) or both (ΔΔ) | **in vitro**: ThT, negative-staining transmission EM, Paramagnetic Relaxation Enhancement NMR, CD  **in vivo** (C. elegans): aggregates formation | -sequence-specific aggregation kinetics compared to WT α-syn  -ΔP1 and ΔΔ reduce aggregation in vivo compared to WT  -P1/P2 sequences affect the function of αSyn in re-modelling lipid vesicles |
| Sun et al.(32)  2021 | WT, E46K and N-terminally acetylated G51D α-Syn | **in vitro**: ThT, negative-staining TEM, AFM, proteolitic digestion assay, Cryo-EM, WB, MALDI-TOF MS  **in cells**: cell viability assay, α-Syn aggregation | -the G51D mutant of α-Syn forms fibrils also in the absence of seed  -the G51D mutant of α-Syn shows a right-handed helical twist  -the G51D mutant of α-Syn reduced stability compared to WT α-Syn  -the G51D mutant of α-Syn induce cross-seeding of WT fibrils  -the G51D mutant of α-Syn show higher toxicity in cells compared to WT |
| Xu et al.(33)  2023 | WT, A30P, E46K, H50Q, A53T and A53E mutants of α-Syn | **in vitro:** α-Syn aggregation, FRAP, turbidity assay, ThT, ThS, Droplet–Droplet Fusion and α-Syn Monomer Recruitment Assay, CD  **in cells:** α-Syn aggregation | -the E46K mutation promotes the LLPS of α-Syn, while the A30P, H50Q, A53T, or A53E mutants have little effect on the initial formation of α-Syn condensates  -none of the mutations influences α-Syn mobility  -all the mutants can cross-react with WT α-Syn  -the A53E mutation retards the rates of liquid-to-solid transition compared to the other mutants  -the E46K mutation promotes the formation of α-Syn condensates in cells |
| Pandit et al.(34)  2023 | WT, S129A and S129W mutants of α-Syn | **in vitro:** MS, CD, ThT, AFM, Raman spectroscopy  **in cells:** α-Syn aggregation, cell viability assay | -both the S129W and the S129A mutations enhance the propensity of helix formation in oligomers compared to WT α-Syn  -both the S129W and the S129A mutations show slower fibrillogenesis compared to WT α-Syn and produce smaller oligomers  -both the S129W and the S129A mutants are less toxic than WT α-Syn in cells when using the results of late time point aggregation as seeds |
| Buratti et al.(35)  2023 | WT and the V15A mutant of α-Syn | **in vitro:** NMR, CD, dynamic light scattering, α-Syn aggregation, EM | -the V15A mutation does not strongly perturb the conformation of monomeric α-Syn in solution  -the V15A α-Syn variant has lower membrane affinity when compared with WT α-Syn  - the V15A α-Syn variant has similar fibrillogenesis compared to WT α-Syn, but it significantly increases in the presence of liopsomes |
| Ohgita et al.(36)  **2023** | WT, A30P, A53T and C-terminally truncated (Δ123‒140 and Δ104‒140) variants of α-Syn | **in vitro:** α-Syn aggregation, ThT, WB, thermodynamic analyses, CD, TEM, TIRFM | - A53T, Δ123‒140, and Δ104‒140 variants increase fibrillogenesis compared to WT α-Syn, while the A20P mutant has less effect  -C-terminal truncations significantly enhance both nucleation and fibril growth, whereas the A53T mutation only enhances the nucleation phase  -variants of α-Syn promote the conversion of monomers to amyloid fibrils at equilibrium compared to WT α-Syn  -truncation of α-Syn induces the formation of β-sheets and fibril formation  -at physiological concentration of α-Syn (the Δ104‒140 truncation enhances also secondary nucleation  -C-terminal truncations have crucial effects on the thermodynamic properties of nucleation and fibril growth of α-Syn |
| Authors  Year | **Tau** | **Settings** | **Highlights** |
| Nacharaju et al. (37)  1999 | Recombinant WT (P301L, R406W, V337M) and mutant human Tau | **in vitro**: heparin or arachidonic acid-induced aggregation, EM | -different mutants display different kinetics of aggregation in the presence of polymerization-inducing agents  -P301L mutant has the highest initial rate of polymerization |
| Rizzini et al.(38)  2000 | Tissue from patient carrying the K257T mutation | **in vitro:** MTs assembly, Tau aggregation, EM | -K257T mutation reduces microtubule assembly  -K257T mutation induces a higher aggregation of 3R but not 4R Tau |
| Neumann et al.(39)  2001 | Recombinant 3R and 4R Tau, both WT and with the K369I mutation | **in vitro:** MTs assembly, Tau aggregation, EM | -K369I mutants show reduced ability to promote microtubule assembly compared to WT Tau  -the 3R K369I mutant gives origin to twisted filaments  -the 4R K369I mutant gives origin to straight filaments  -both 3R and 4R K369I mutants produce only small fragments of filaments |
| Grover et al.(40)  2003 | K257T, I260V mutations of 2N/3R and 2N/4R and 2N/4R Tau containing the ΔK280 mutation | **in vitro**: Tau aggregation, ThS, MTs assembly | -the I260V mutation induces significantly higher aggregation in the 2N/4R Tau but not in the 2N/3R Tau (compared to WT  -K257T mutation did not affect significantly the aggregation of Tau compared to WT  -both I260V and K257T mutations cause a decrease in tubulin assembly |
| Pickering-Brown et al.(41)  2004 | Tissue from patient carrying the Q336R mutation | **in vitro:** MTs assembly, Tau aggregation, EM  **ex vivo**: neuropathological examination | -the Q336R of both 3R and 4R Tau promotes microtubule assembly  -the Q336R mutation promotes aggregation of Tau, which is more prominent for the 3R form |
| Neumann et al.(42)  2005 | G335V mutation of 3R and 4R Tau | **in vitro:** MTs assembly, Tau aggregation | -the G335V mutation reduce microtubule assembly compared to WT  -the G335V mutation induce a higher aggregation of Tau compared to WT |
| Van Swieten et al.(43)  2007 | Tissue from patient carrying the ΔK280 mutation | **in vitro:** Tau extraction, EM  **ex vivo**: neuropathological examination | -the ΔK280 mutation induce a higher 3R/4R ratio in brain tissue |
| Chang et al.(44)  2008 | Recombinant 2N/4R Tau WT, and mutants R5L, G272V, P301L, V337M, and R406W | **in vitro:** Thiazine red-induced aggregation | -the G272V and P301L mutations promote Tau aggregation  -the R5L, G272V, P301L, and V337M mutations accelerate the nucleation phase |
| Jeganathan et al.(45)  2008 | WT and phosphomutants of the full-length Tau isoform hTau40 (4R/2N) | **in vitro:** FRET, CD Spectroscopy, Tau aggregation, MTs assembly, WB | -combination of pseudo-phosphorylated epitopes affects Tau folding and aggregation, but does not affect MTs assembly |
| Alonso et al(46)  2010 | Phosphomutants of WT Tau and R406W Tau | **in cells:** ICC, overlay assays | -the phosphorylation at residues Thr^212^, Ser^235^, and Ser^262^ reduce the ability of Tau to bind microtubules  -Thr^212^ phosphorylation can induce aggregation of R406W Tau  -phosphorylation at Thr^212^, Thr^231^, and Ser^262^ promote Tau toxicity |
| Bibow et al.(47)  2011 | R2/wt (Ac–GKVQIINKKLDL–NH2) and R2/ΔK280 Tau (Ac–GKVQIINKLDL–NH2) | **in vitro:** MS and ion mobility (IM-MS), TEM, ThT | -R2/ΔK280 aggregates more rapidly than R2/wt in the presence of heparin  -The removal of this constraint in R2/ΔK280 leads to more extended structures, which are more prone to aggregation |
| Combs and Gamblin (48)  2012 | 12 mutants and WT | **in vitro**: Tht, Right-angle laser light scattering, TEM | -different mutants have different effects on Tau polymerization, aggregate morphology, kinetics of polymerization and microtubule assembly |
| Iyer et al.(49)  2013 | Recombinant 3R/2N Tau and 4R/2N Tau WT and G55R | **in vitro:** MTs assembly, Tau aggregation | -4R G55R mutant Tau nucleates and assembles MTs more effectively than 4R and 3R Tau WT and of the 3R G55R mutant, although assembled MTs are shorter  -the G55R mutation does not affect MTs dynamics  --the G55R mutation does not affect Tau aggregation |
| Rossi et al.(50)  2014 | Recombinant 2N/4R WT, V363I, V363A and P301L mutations | **in vitro**: laser light scattering, Turbidimetry | - V363A Tau shows a reduced ability to promote microtubule assembly compared to WT, but similar to the P301L mutant  -V363I Tau shows a greater ability to promote microtubule assembly compared to WT  -Both V363A and V363I show reduced fibrillogenesis (but high formation of oligomers) |
| Raz et al.(51)  2014 | WT and ΔK280 repeat R2 oligomers | **in vitro:** ssNMR, TEM, AFM | -the ΔK280 mutation in the R2 repeat increases the formation of small aggregates (oligomers) and fewer fibrils compared to WT  -an extension of the ΔK280 mutant by one residue in the N- or the C-terminus leads to more fibril formation  -an extension of the ΔK280 mutant by one residue in the N- or the C-terminus leads to polymorphism. |
| Ferreon et al.(52)  2018 | WT Hyperacetylated Tau (Ac-Tau) 2N4/R Tau | **in vitro**: ThT, MTs assembly, MS | -hyperacetylation of Tau disfavors liquid-liquid phase separation  -Ac-Tau reduces heparin-induced aggregation of Tau  -Ac-Tau reduces fibrillogenesis of Tau  -AC-Tau has reduced ability to promote MTs assembly |
| Strang et al.(53)  2018 | 15 mutants and WT (0N/4R and 2N/4R): inoculation with WT or mutant K18 Tau fibrils | **in cells**: Tau, ThS staining, EM | -different mutants have distinct aggregation propensity  -the presence of a Pro residue at position 301 is crucial to inhibit Tau aggregation |
| Morelli et al.(54)  2018 | C. Elegans expressing human Tau WT , Tau V363I and Tau V363A under the control of aex-3 neuronal  promoter | **in vitro:** WB  **in vivo:** behavioural studies | -The V363I mutants present less soluble Tau compared to WT and to V363A mutants  - The V363I mutants present more phosphorylated Tau compared to WT and to V363A mutants  -The V363A mutants have higher propensity for the formation of Tau oligomers. |
| Strang et al.(55)  2019 | 0N/4R and 2N4/R human Tau isoforms and phosphomimetics; K18 peptide | **in vitro:** WB | -The presence of the S305E phosphomimetic significantly reduced aggregation of Tau |
| Mutreja et al.(56)  2019 | Recombinant WT and R5L, P301L, and R406W mutants of the 4R isoforms | **in vitro:** Tau aggregation, ThS, Right-Angle Laser Light Scattering Assay, TEM  **in cells:** ThS, MTs assembly | -the 3 mutants specifically affect the extent of aggregation, the kinetics of aggregation, the morphology of aggregates and the polymerization of MTs |
| Carlomagno et al.(57)  2019 | Mice injected with V5-tagged Tau^A152T^ or V5-tagged Tau^P301L^ | **in vitro:** WB, IHC  **in vivo:** intracerebroventricular injections | -P301L and A152T lead to a very different pattern of Tau deposition in vivo  -A152T mutation slows aggregation of hyperphosphorylated Tau |
| Rane et al.(58)  2019 | WT, K274Q (acetylmimetic)and K274R mutants of Tau | **in vitro:** Tau aggregation, dot blot, EM, AFM, CD, dynamic light scattering, surface plasmon resonance, ThS  **in cells**: Tau internalization, MTs binding | -Acetylation at K274 (K274Q) changes the secondary structure of Tau  -K274Q Tau binds to MTs with lower affinity than WT and K274R Tau and has a reduce ability to induce MTs polymerization in cells  -the propensity of K273Q Tau to aggregate and form droplets is higher than both WT and K274R Tau  -K274Q Tau formed significantly more and larger size oligomers than WT Tau  -K274Q Tau induce more cytotoxicity than WT Tau |
| Karikari et al.(59)  2019 | WT and V337M and N279K mutants of K18 fragment | **in vitro:** Tau aggregation, dot blot, TEM, ICC.  **in cells:** Tau aggregation, confocal microscopy, LDH assay | -WT and V337M and N279K mutants differ as for the aggregates ultra-structure  -all the variants show different immune-reactivity  -both V337M and N279K mutants promote Tau internalization in SH-SY5Y cells |
| Karikari et al.(60)  2020 | WT and C291R K18 fragment | **in vitro:** CD, Spectroscopy, Tau aggregation, AFM | -the C291R variant has a higher propensity to adopt β-sheet conformation  -compared to WT, the C291R mutation promotes the formation of non-fibrillar oligomers and amorphous assemblies |
| Kawasaki et al.(61)  2020 | WT and TauF4Δ fragment (225–324 aa) | **in vitro:** NMR, NMR spin relaxation, Paramagnetic Relaxation Enhancement, Paramagnetic Relaxation Interference | -the P301L mutation does not change conformation dynamics on the ns timescale  -the P301L mutation destabilizes the transient folding β-structure of the β3 part while not affecting the dynamic β-structures of PHF6* and PHF6, leading to a more extended conformation  - The P301L mutation caused small but clear changes in local and long-range correlations  - the P301L mutation exclusively affects transient forms of TauF4Δ |
| Shimonaka et al.(62)  2020 | WT and 10 different deletion mutants of Tau-CTF24 (243–441 aa) lacking 16-amino acid residues | **in vitro:** Tau aggregation with different seeds, ICC, ThS, MTs assembly, WB, TEM  **in cells:** Tau aggregation, | -Δ306–321 aa, Δ322–337 aa and Δ353–368 aa mutants reduce insoluble Tau in SH-SY5Y cells treated with different seeds  -Δ275–290 aa, Δ306–321 aa, Δ353–368 aa and Δ369–384 aa mutants reduce the aggregation of endogenous Tau in SH-SY5Y cells treated with different seeds  -Δ306–321 aa and Δ353–368 aa mutants show decrease in ThS fluorescence compared with the WT  -deletion of Asn-368 and S320A decrease Tau aggregation using AD Tau seed  -deletion of the Asn-368 residue affects the MT-binding ability of Tau |
| Sandberg et al.(63)  2020 | transgenic Drosophila overexpressing 0N/4R and 0N/3R Tau, both WT and G273R mutants | **in vitro:** Tau aggregation, TEM, MTs and F-actin binding assays  **in vivo:** amyloid aggregate detection | -the 0N/4R G273R promote aggregation in glial cells in Drosophila  -the 0N/4R G273R mutant aggregates faster than WT and than 0N/3R variants  -the G273R mutation affects fibrils structure  -the 0N/3R G273R mutant shows the highest affinity for F-actin |
| Siano et al.(64)  2020 | WT and Q336H mutant of Tau | **in vitro:** Tau aggregation, IF, FRET, FRAP, Tau aggregation  **in cells**: Tau aggregation | -the Q336H mutant alters Tau conformation  -the Q336H mutation determines a stronger and more stable interaction with tubulin compared to WT  -the Q336H mutation reduces Tau phosphorylation  -the Q336H mutation increases MTs polymerization  -the Q336H mutation increase Tau aggregation in HeLa cells |
| Losev et al. (65)  2021 | Transgenic Drosophila, expressing WT or the  N167Q, N359Q, N410Q human Tau mutants | **in vitro:** MS/MS analysis  **in cells:** SH-SY5Y cells transfection, WB  **in vivo:** IHC, SEM, behavioural analyses | -N359 and N410 undergo N‑glycosylation in human Tau  expressed in cells  -N167Q and N359Q mutants reduce Tau phosphorylation, while  the N410Q reduces total Tau level  -N-glycosylation at N410 modulates phosphorylation at S409  -N167Q and N359Q mutants reduce accumulation of Tau in fly discs, while N410Q mutant increases the accumulation  -N359Q mutant ameliorate eye phenotype and climbing activity and increase life span of flies, while N410Q worsen both the eye phenotype and the climbing activity of flies |
| Kumar et al.(66)  2021 | WT, K280Q (acetylmimetic) and K280E human Tau K18 fragment | **in vitro:** ThT, AFM, Peptide mapping and hydrogen‐exchange MS, Far‐UV circular dichroism, Multi angle light scattering and dynamic light scattering | -the K280Q mutation affects the structure of fibrils  -Fibrils of Tau‐K18 K280Q convert monomeric Tau into fibrils more slowly than fibrils of Tau‐K18  -Tau‐K18 K280Q fibrils are more stable than Tau‐K18 fibrils  -monomeric Tau‐K18 K280Q is converted more rapidly into fibrils than is monomeric Tau‐K18 |
| Xia et al.(67)  2021 | WT 0N/4R Tau and acetylmimetics K259Q, K290Q, K321Q, and K353Q | **in vitro:** WB  **in cells:** Tau aggregation, MTs binding, ThS, IF | -K259Q, K290Q, K321Q, and K353Q all significantly decreased Tau-MT  -K321Q and K353Q acetylmimetics decrease Tau aggregation |
| Xia et al.(68)  2021 | WT and P301L, Q336H, and Q336R of 0N/3R and 0N/4R human Tau | **in vitro:** MTs binding, WB  **in cells:** Tau aggregation, IF | -Q336R but not Q336H 0N/4R and 0N/3RTau significantly aggregated with K18 seeding  -Q336R/P301L but not Q336H/P301L 0N/4R Tau presents enhanced aggregation without seeding  -aggregates of the 0N/3R isoform are more phosphorylated than their 0N/4R isoform counterparts.  -Q336R but not Q336H 0N/4R Tau exhibit increased MT binding affinity compared to WT 0N/4R  -both Q336H and Q336R 0N/3R Tau exhibit increased MT binding affinity compared to WT 0N/3R  -All the mutant show decreased levels of tyrosinated MTs compared to cells expressing WT Tau  - Q336R 0N/3R Tau presents decreased acetylated tubulin compared to WT |
| De Luigi et al.(69)  2022 | 2N/4R WT, P301L, V363A, and V363I mutants | **in vitro:** CD, Small-angle X-ray scattering, Tau aggregation, ThT, AFM  **in cells**: toxicity assay | -none of the 3 mutations affect folding of monomeric Tau in solution  -P301L shows the higher propensity to form β-sheet structures and fibrillogenesis kinetics, followed by WT and the two V363 mutations  -V363A and V363I mutants have intermediate toxicity compared to WT and P301L Tau |
| Chen et al.(70)  2023 | 4 R TauRD (aa 243–380), 3 R TauRD (aa 243–380 missing 275–305) and FL 2N/4R Tau WT, S320F and P301S mutants | **in vitro:** ThT, TEM, MS, Molecular dynamics simulations  **in cells:** Tau aggregation, FRET | -The S320F mutantaggregates spontaneously in vitro and in cells  -The S320F mutation changes the clustering of nonpolar residues in the monomer towards a pro-aggregation conformation  -The I328S mutation prevents S320F-induced Tau aggregation both in vitro and in cells |
| Xia et al.(71)  2023 | WT 0N/4R Tau and methylmimetics: K174F, K180F, K254F, K267F, K369F | **in vitro:** WB  **in cells:** MTs binding, Tau aggregation, | -K174F, K267F and K369F Tau show decreased MT binding relative to WT Tau  -Tau methylmimetics K174F, K180F, K254F, K267F, and K369F alone do not change aggregation rate in cells compared to WT Tau but enhance P301L-mediated aggregation |
| Xia et al.(72)  2023 | 0N/3R human Tau WT and G335V, G366R, S356T, V363I, S352L and V363A mutants | **in vitro:** MTs binding, WB,  **in cells:** Tau aggregation, IF, ThS | -G335V and G366R mutants display reduced MT binding compared to WT Tau  -S356T and V363I mutants display increased MT binding compared to WT Tau  -S352L and V363A mutants show MT binding similar to WT Tau  -mutants G335V, S352L andV363A did not significantly aggregate with or without K19 seeds  -V363I and G366R have modest intrinsic aggregate both in the absence and in the presence of K19 seeds  -S356T 0N3R Tau aggregate only when treated with K19 seeds both in vitro and in cells |
| Abbreviations: AFM: Atomic Force Microscopy, CD: Circular Dichroism, EM: Electron microscopy, FACS: Fluorescence-activated cell sorting, FC: Flow cytometry, FRAP: Fluorescence Recovery After Photo bleaching, FRET: Fluorescence Resonance Energy Transfer, FTIR: Fourier Transform Infrared Spectroscopy, ICC: immunocytochemistry, IF: immunofluorescence, IHC: immunohistochemistry, LDH: lactate dehydrogenase, MS: mass spectrometry, MTs: microtubules, NMR: Nuclear Magnetic Resonance, SEC: Size Exclusion Chromatography, SEM: Scanning Electron Microscopy, TEM: Transmission Electron Microscopy, ThS: Thioflavin S, ThT: Thioflavin T, TIRFM: Total Internal Reflection Fluorescence Microscopy, VCD: Vibrational Circular Dichroism, WB: Western Blot | | | |

**References:**

1. Conway KA, Harper JD, Lansbury PT. Accelerated in vitro fibril formation by a mutant alpha-synuclein linked to early-onset Parkinson disease. Nat Med. 1998;4(11):1318-20.

2. Narhi L, Wood SJ, Steavenson S, Jiang Y, Wu GM, Anafi D, et al. Both familial Parkinson's disease mutations accelerate alpha-synuclein aggregation. J Biol Chem. 1999;274(14):9843-6.

3. Ostrerova-Golts N, Petrucelli L, Hardy J, Lee JM, Farer M, Wolozin B. The A53T alpha-synuclein mutation increases iron-dependent aggregation and toxicity. J Neurosci. 2000;20(16):6048-54.

4. Li J, Uversky VN, Fink AL. Effect of familial Parkinson's disease point mutations A30P and A53T on the structural properties, aggregation, and fibrillation of human alpha-synuclein. Biochemistry. 2001;40(38):11604-13.

5. Lee MK, Stirling W, Xu Y, Xu X, Qui D, Mandir AS, et al. Human alpha-synuclein-harboring familial Parkinson's disease-linked Ala-53 --> Thr mutation causes neurodegenerative disease with alpha-synuclein aggregation in transgenic mice. Proc Natl Acad Sci U S A. 2002;99(13):8968-73.

6. Murray IV, Giasson BI, Quinn SM, Koppaka V, Axelsen PH, Ischiropoulos H, et al. Role of alpha-synuclein carboxy-terminus on fibril formation in vitro. Biochemistry. 2003;42(28):8530-40.

7. Zhou W, Freed CR. Tyrosine-to-cysteine modification of human alpha-synuclein enhances protein aggregation and cellular toxicity. J Biol Chem. 2004;279(11):10128-35.

8. Li W, West N, Colla E, Pletnikova O, Troncoso JC, Marsh L, et al. Aggregation promoting C-terminal truncation of alpha-synuclein is a normal cellular process and is enhanced by the familial Parkinson's disease-linked mutations. Proc Natl Acad Sci U S A. 2005;102(6):2162-7.

9. Pandey N, Schmidt RE, Galvin JE. The alpha-synuclein mutation E46K promotes aggregation in cultured cells. Exp Neurol. 2006;197(2):515-20.

10. Meier F, Abeywardana T, Dhall A, Marotta NP, Varkey J, Langen R, et al. Semisynthetic, site-specific ubiquitin modification of alpha-synuclein reveals differential effects on aggregation. J Am Chem Soc. 2012;134(12):5468-71.

11. Marotta NP, Cherwien CA, Abeywardana T, Pratt MR. O-GlcNAc modification prevents peptide-dependent acceleration of alpha-synuclein aggregation. Chembiochem. 2012;13(18):2665-70.

12. Ghosh D, Mondal M, Mohite GM, Singh PK, Ranjan P, Anoop A, et al. The Parkinson's disease-associated H50Q mutation accelerates alpha-Synuclein aggregation in vitro. Biochemistry. 2013;52(40):6925-7.

13. Bousset L, Pieri L, Ruiz-Arlandis G, Gath J, Jensen PH, Habenstein B, et al. Structural and functional characterization of two alpha-synuclein strains. Nat Commun. 2013;4:2575.

14. Khalaf O, Fauvet B, Oueslati A, Dikiy I, Mahul-Mellier AL, Ruggeri FS, et al. The H50Q mutation enhances alpha-synuclein aggregation, secretion, and toxicity. J Biol Chem. 2014;289(32):21856-76.

15. Fares MB, Ait-Bouziad N, Dikiy I, Mbefo MK, Jovicic A, Kiely A, et al. The novel Parkinson's disease linked mutation G51D attenuates in vitro aggregation and membrane binding of alpha-synuclein, and enhances its secretion and nuclear localization in cells. Hum Mol Genet. 2014;23(17):4491-509.

16. Ghosh D, Sahay S, Ranjan P, Salot S, Mohite GM, Singh PK, et al. The newly discovered Parkinson's disease associated Finnish mutation (A53E) attenuates alpha-synuclein aggregation and membrane binding. Biochemistry. 2014;53(41):6419-21.

17. Lazaro DF, Rodrigues EF, Langohr R, Shahpasandzadeh H, Ribeiro T, Guerreiro P, et al. Systematic comparison of the effects of alpha-synuclein mutations on its oligomerization and aggregation. PLoS Genet. 2014;10(11):e1004741.

18. Rutherford NJ, Moore BD, Golde TE, Giasson BI. Divergent effects of the H50Q and G51D SNCA mutations on the aggregation of alpha-synuclein. J Neurochem. 2014;131(6):859-67.

19. Xiang W, Menges S, Schlachetzki JC, Meixner H, Hoffmann AC, Schlotzer-Schrehardt U, et al. Posttranslational modification and mutation of histidine 50 trigger alpha synuclein aggregation and toxicity. Mol Neurodegener. 2015;10:8.

20. Rutherford NJ, Giasson BI. The A53E alpha-synuclein pathological mutation demonstrates reduced aggregation propensity in vitro and in cell culture. Neurosci Lett. 2015;597:43-8.

21. Marotta NP, Lin YH, Lewis YE, Ambroso MR, Zaro BW, Roth MT, et al. O-GlcNAc modification blocks the aggregation and toxicity of the protein alpha-synuclein associated with Parkinson's disease. Nat Chem. 2015;7(11):913-20.

22. Mason RJ, Paskins AR, Dalton CF, Smith DP. Copper Binding and Subsequent Aggregation of alpha-Synuclein Are Modulated by N-Terminal Acetylation and Ablated by the H50Q Missense Mutation. Biochemistry. 2016;55(34):4737-41.

23. Flagmeier P, Meisl G, Vendruscolo M, Knowles TP, Dobson CM, Buell AK, et al. Mutations associated with familial Parkinson's disease alter the initiation and amplification steps of alpha-synuclein aggregation. Proc Natl Acad Sci U S A. 2016;113(37):10328-33.

24. Lazaro DF, Dias MC, Carija A, Navarro S, Madaleno CS, Tenreiro S, et al. The effects of the novel A53E alpha-synuclein mutation on its oligomerization and aggregation. Acta Neuropathol Commun. 2016;4(1):128.

25. Inigo-Marco I, Valencia M, Larrea L, Bugallo R, Martinez-Goikoetxea M, Zuriguel I, et al. E46K alpha-synuclein pathological mutation causes cell-autonomous toxicity without altering protein turnover or aggregation. Proc Natl Acad Sci U S A. 2017;114(39):E8274-E83.

26. Iyer A, Roeters SJ, Kogan V, Woutersen S, Claessens M, Subramaniam V. C-Terminal Truncated alpha-Synuclein Fibrils Contain Strongly Twisted beta-Sheets. J Am Chem Soc. 2017;139(43):15392-400.

27. Afitska K, Fucikova A, Shvadchak VV, Yushchenko DA. Modification of C Terminus Provides New Insights into the Mechanism of alpha-Synuclein Aggregation. Biophys J. 2017;113(10):2182-91.

28. de Oliveira GAP, Silva JL. Alpha-synuclein stepwise aggregation reveals features of an early onset mutation in Parkinson's disease. Commun Biol. 2019;2:374.

29. Boyer DR, Li B, Sun C, Fan W, Sawaya MR, Jiang L, et al. Structures of fibrils formed by alpha-synuclein hereditary disease mutant H50Q reveal new polymorphs. Nat Struct Mol Biol. 2019;26(11):1044-52.

30. Zhao K, Li Y, Liu Z, Long H, Zhao C, Luo F, et al. Parkinson's disease associated mutation E46K of alpha-synuclein triggers the formation of a distinct fibril structure. Nat Commun. 2020;11(1):2643.

31. Doherty CPA, Ulamec SM, Maya-Martinez R, Good SC, Makepeace J, Khan GN, et al. A short motif in the N-terminal region of alpha-synuclein is critical for both aggregation and function. Nat Struct Mol Biol. 2020;27(3):249-59.

32. Sun Y, Long H, Xia W, Wang K, Zhang X, Sun B, et al. The hereditary mutation G51D unlocks a distinct fibril strain transmissible to wild-type alpha-synuclein. Nat Commun. 2021;12(1):6252.

33. Xu B, Fan F, Liu Y, Liu Y, Zhou L, Yu H. Distinct Effects of Familial Parkinson's Disease-Associated Mutations on alpha-Synuclein Phase Separation and Amyloid Aggregation. Biomolecules. 2023;13(5).

34. Pandit E, Das L, Das AK, Dolui S, Saha S, Pal U, et al. Single point mutations at the S129 residue of alpha-synuclein and their effect on structure, aggregation, and neurotoxicity. Front Chem. 2023;11:1145877.

35. Buratti FA, Fernandez CO, Zweckstetter M. Parkinson's disease-linked V15A mutation facilitates alpha-synuclein aggregation by reducing membrane affinity. Protein Sci. 2023;32(8):e4693.

36. Ohgita T, Namba N, Kono H, Shimanouchi T, Saito H. Mechanisms of enhanced aggregation and fibril formation of Parkinson's disease-related variants of alpha-synuclein. Sci Rep. 2022;12(1):6770.

37. Nacharaju P, Lewis J, Easson C, Yen S, Hackett J, Hutton M, et al. Accelerated filament formation from tau protein with specific FTDP-17 missense mutations. FEBS Lett. 1999;447(2-3):195-9.

38. Rizzini C, Goedert M, Hodges JR, Smith MJ, Jakes R, Hills R, et al. Tau gene mutation K257T causes a tauopathy similar to Pick's disease. J Neuropathol Exp Neurol. 2000;59(11):990-1001.

39. Neumann M, Schulz-Schaeffer W, Crowther RA, Smith MJ, Spillantini MG, Goedert M, et al. Pick's disease associated with the novel Tau gene mutation K369I. Ann Neurol. 2001;50(4):503-13.

40. Grover A, England E, Baker M, Sahara N, Adamson J, Granger B, et al. A novel tau mutation in exon 9 (1260V) causes a four-repeat tauopathy. Exp Neurol. 2003;184(1):131-40.

41. Pickering-Brown SM, Baker M, Nonaka T, Ikeda K, Sharma S, Mackenzie J, et al. Frontotemporal dementia with Pick-type histology associated with Q336R mutation in the tau gene. Brain. 2004;127(Pt 6):1415-26.

42. Neumann M, Diekmann S, Bertsch U, Vanmassenhove B, Bogerts B, Kretzschmar HA. Novel G335V mutation in the tau gene associated with early onset familial frontotemporal dementia. Neurogenetics. 2005;6(2):91-5.

43. van Swieten JC, Bronner IF, Azmani A, Severijnen LA, Kamphorst W, Ravid R, et al. The DeltaK280 mutation in MAP tau favors exon 10 skipping in vivo. J Neuropathol Exp Neurol. 2007;66(1):17-25.

44. Chang E, Kim S, Yin H, Nagaraja HN, Kuret J. Pathogenic missense MAPT mutations differentially modulate tau aggregation propensity at nucleation and extension steps. J Neurochem. 2008;107(4):1113-23.

45. Jeganathan S, Hascher A, Chinnathambi S, Biernat J, Mandelkow EM, Mandelkow E. Proline-directed pseudo-phosphorylation at AT8 and PHF1 epitopes induces a compaction of the paperclip folding of Tau and generates a pathological (MC-1) conformation. J Biol Chem. 2008;283(46):32066-76.

46. Alonso AD, Di Clerico J, Li B, Corbo CP, Alaniz ME, Grundke-Iqbal I, et al. Phosphorylation of tau at Thr212, Thr231, and Ser262 combined causes neurodegeneration. J Biol Chem. 2010;285(40):30851-60.

47. Bibow S, Ozenne V, Biernat J, Blackledge M, Mandelkow E, Zweckstetter M. Structural impact of proline-directed pseudophosphorylation at AT8, AT100, and PHF1 epitopes on 441-residue tau. J Am Chem Soc. 2011;133(40):15842-5.

48. Combs B, Gamblin TC. FTDP-17 tau mutations induce distinct effects on aggregation and microtubule interactions. Biochemistry. 2012;51(43):8597-607.

49. Iyer A, Lapointe NE, Zielke K, Berdynski M, Guzman E, Barczak A, et al. A novel MAPT mutation, G55R, in a frontotemporal dementia patient leads to altered Tau function. PLoS One. 2013;8(9):e76409.

50. Rossi G, Bastone A, Piccoli E, Morbin M, Mazzoleni G, Fugnanesi V, et al. Different mutations at V363 MAPT codon are associated with atypical clinical phenotypes and show unusual structural and functional features. Neurobiol Aging. 2014;35(2):408-17.

51. Raz Y, Adler J, Vogel A, Scheidt HA, Haupl T, Abel B, et al. The influence of the DeltaK280 mutation and N- or C-terminal extensions on the structure, dynamics, and fibril morphology of the tau R2 repeat. Phys Chem Chem Phys. 2014;16(17):7710-7.

52. Ferreon JC, Jain A, Choi KJ, Tsoi PS, MacKenzie KR, Jung SY, et al. Acetylation Disfavors Tau Phase Separation. Int J Mol Sci. 2018;19(5).

53. Strang KH, Croft CL, Sorrentino ZA, Chakrabarty P, Golde TE, Giasson BI. Distinct differences in prion-like seeding and aggregation between Tau protein variants provide mechanistic insights into tauopathies. J Biol Chem. 2018;293(7):2408-21.

54. Morelli F, Romeo M, Barzago MM, Bolis M, Mattioni D, Rossi G, et al. V363I and V363A mutated tau affect aggregation and neuronal dysfunction differently in C. elegans. Neurobiol Dis. 2018;117:226-34.

55. Strang KH, Sorrentino ZA, Riffe CJ, Gorion KM, Vijayaraghavan N, Golde TE, et al. Phosphorylation of serine 305 in tau inhibits aggregation. Neurosci Lett. 2019;692:187-92.

56. Mutreja Y, Combs B, Gamblin TC. FTDP-17 Mutations Alter the Aggregation and Microtubule Stabilization Propensity of Tau in an Isoform-Specific Fashion. Biochemistry. 2019;58(6):742-54.

57. Carlomagno Y, Chung DC, Yue M, Kurti A, Avendano NM, Castanedes-Casey M, et al. Enhanced phosphorylation of T153 in soluble tau is a defining biochemical feature of the A152T tau risk variant. Acta Neuropathol Commun. 2019;7(1):10.

58. Rane JS, Kumari A, Panda D. An acetylation mimicking mutation, K274Q, in tau imparts neurotoxicity by enhancing tau aggregation and inhibiting tubulin polymerization. Biochem J. 2019;476(10):1401-17.

59. Karikari TK, Nagel DA, Grainger A, Clarke-Bland C, Crowe J, Hill EJ, et al. Distinct Conformations, Aggregation and Cellular Internalization of Different Tau Strains. Front Cell Neurosci. 2019;13:296.

60. Karikari TK, Thomas R, Moffat KG. The C291R Tau Variant Forms Different Types of Protofibrils. Front Mol Neurosci. 2020;13:39.

61. Kawasaki R, Tate SI. Impact of the Hereditary P301L Mutation on the Correlated Conformational Dynamics of Human Tau Protein Revealed by the Paramagnetic Relaxation Enhancement NMR Experiments. Int J Mol Sci. 2020;21(11).

62. Shimonaka S, Matsumoto SE, Elahi M, Ishiguro K, Hasegawa M, Hattori N, et al. Asparagine residue 368 is involved in Alzheimer's disease tau strain-specific aggregation. J Biol Chem. 2020;295(41):13996-4014.

63. Sandberg A, Ling H, Gearing M, Dombroski B, Cantwell L, R'Bibo L, et al. Fibrillation and molecular characteristics are coherent with clinical and pathological features of 4-repeat tauopathy caused by MAPT variant G273R. Neurobiol Dis. 2020;146:105079.

64. Siano G, Micaelli M, Scarlatti A, Quercioli V, Di Primio C, Cattaneo A. The Q336H MAPT Mutation Linked to Pick's Disease Leads to Increased Binding of Tau to the Microtubule Network via Altered Conformational and Phosphorylation Effects. Front Mol Neurosci. 2020;13:569395.

65. Losev Y, Frenkel-Pinter M, Abu-Hussien M, Viswanathan GK, Elyashiv-Revivo D, Geries R, et al. Differential effects of putative N-glycosylation sites in human Tau on Alzheimer's disease-related neurodegeneration. Cell Mol Life Sci. 2021;78(5):2231-45.

66. Kumar H, Udgaonkar JB. The Lys 280 --> Gln mutation mimicking disease-linked acetylation of Lys 280 in tau extends the structural core of fibrils and modulates their catalytic properties. Protein Sci. 2021;30(4):785-803.

67. Xia Y, Bell BM, Giasson BI. Tau K321/K353 pseudoacetylation within KXGS motifs regulates tau-microtubule interactions and inhibits aggregation. Sci Rep. 2021;11(1):17069.

68. Xia Y, Nasif L, Giasson BI. Pathogenic MAPT mutations Q336H and Q336R have isoform-dependent differences in aggregation propensity and microtubule dysfunction. J Neurochem. 2021;158(2):455-66.

69. De Luigi A, Colombo L, Russo L, Ricci C, Bastone A, Cimini S, et al. Biochemical and biophysical features of disease-associated tau mutants V363A and V363I. Biochim Biophys Acta Proteins Proteom. 2022;1870(3):140755.

70. Chen D, Bali S, Singh R, Wosztyl A, Mullapudi V, Vaquer-Alicea J, et al. FTD-tau S320F mutation stabilizes local structure and allosterically promotes amyloid motif-dependent aggregation. Nat Commun. 2023;14(1):1625.

71. Xia Y, Bell BM, Giasson BI. Tau Lysine Pseudomethylation Regulates Microtubule Binding and Enhances Prion-like Tau Aggregation. Int J Mol Sci. 2023;24(9).

72. Xia Y, Bell BM, Kim JD, Giasson BI. Tau mutation S356T in the three repeat isoform leads to microtubule dysfunction and promotes prion-like seeded aggregation. Front Neurosci. 2023;17:1181804.
